# Outcomes After Kidney Transplantation in Antineutrophil Cytoplasmic Autoantibody–Associated Renal Vasculitis

**DOI:** 10.1016/j.ekir.2025.07.008

**Published:** 2025-07-14

**Authors:** Marine Dekervel, Priscille Traversat, Philippe Gatault, Léonard Golbin, Antoine Thierry, Valérie Chatelet, Sophie Caillard, Anna Duval, Emilie Cornec-Le Gall, Martin Planchais, Agnès Duveau, Dominique Bertrand, Jean-Philippe Rerolle, Cyrille Garrouste, Dany Anglicheau, Maïté Jaureguy, Fabien Duthe, Jean-François Augusto, Benoit Brilland

**Affiliations:** 1 Service de Néphrologie-Dialyse-Transplantation, CHU Angers, Angers, France; 2 Service de Néphrologie-Dialyse-Transplantation, CHU de Tours, Tours, France; 3 Service de Néphrologie-Dialyse-Transplantation, CHU de Rennes, Rennes, France; 4 Service de Néphrologie-Dialyse-Transplantation, CHU de Poitiers, Poitiers, France; 5 Service de Néphrologie-Dialyse-Transplantation, CHU de Caen, Caen, France; 6 Service de Néphrologie-Dialyse-Transplantation, CHU de Strasbourg, Strasbourg, France; 7 Université de Brest, Inserm, UMR 1078, GGB, CHU Brest, Centre de référence MARHEA, Brest, France; 8 Service de Néphrologie-Dialyse-Transplantation, CHU de Rouen, Rouen, France; 9 Service de Néphrologie-Dialyse-Transplantation, CHU de Limoges, Limoges, France; 10 Service de Néphrologie-Dialyse-Transplantation, CHU de Clermont-Ferrand, Clermont-Ferrand, France; 11 Service des Maladies du rein et du métabolisme, , Hôpital Necker, Assistance Publique-Hôpitaux de Paris, Paris, France; 12 Service de Néphrologie-Dialyse-Transplantation, CHU d’Amiens, Amiens, France; 13 Univ Angers, Nantes Université, Inserm, CNRS, CRCI2NA, SFR ICAT, Angers, France

**Keywords:** ANCA, glomerulonephritis, kidney transplantation, outcomes, vasculitis

## Abstract

**Introduction:**

Antineutrophil cytoplasmic autoantibody (ANCA)-associated renal vasculitis (AAV) glomerulonephritis (GN) (AAV-GN) frequently leads to end-stage kidney disease (ESKD). Optimal management for patients with AAV-GN receiving a kidney transplantation (KT) remains poorly characterized. We compared posttransplant outcomes between patients with AAV-GN and controls in the modern era of immunosuppressive therapy.

**Methods:**

This multicenter retrospective study included 206 adult patients with AAV-GN and 412 matched controls who underwent KT between 2005 and 2023 in 12 French transplant centers. We compared the incidence of delayed graft function (DGF), graft failure, AAV relapses, acute rejection, and mortality between groups; and analyzed risk factors using multivariable models.

**Results:**

DGF incidence and kidney function up to 5 years after KT were similar between groups. Patients with AAV-GN showed a trend toward poorer graft survival (hazard ratio [HR] = 1.55, *P* = 0.077) and significantly lower overall survival (HR = 1.48, *P* = 0.034). AAV relapses occurred in 15 patients with AAV-GN (7.3%), significantly impacting graft survival (*P* = 0.008). ANCA positivity at KT tended to be associated with higher relapse risk (HR = 4.17, *P* = 0.065) and was associated with lower rejection risk (HR = 0.31, *P* = 0.016). Acute rejection incidence was comparable between groups. Azathioprine (AZA) maintenance therapy was associated with increased rejection (HR = 3.733, *P* = 0.012) and graft failure (HR = 3.73, *P* = 0.007 < 0.001). Although patients with AAV-GN were waitlisted later than controls, no specific transplant timing was associated with improved outcomes.

**Conclusion:**

Although KT offers patients with AAV-GN short-term outcomes similar to controls, they face higher long-term risk of graft failure and mortality. ANCA status at transplantation may help predict immunological events, emphasizing the need for careful evaluation and monitoring, but without delaying the process. As for other nephropathies, AZA should be avoided as maintenance therapy in patients with AAV-GN. These findings highlight the need for tailored posttransplant management in patients with AAV-GN.

## Graphical abstract

**Figure:**
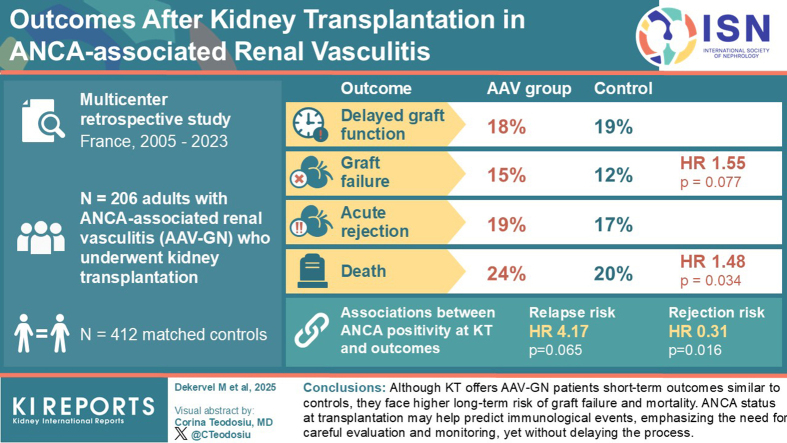

See Commentary on Page 3300

Kidney involvement in AAV is very frequent. Usually presenting as a rapidly progressive GN (i.e., AAV-GN), it is found in 80% to 100% of microscopic polyangiitis, and 60% to 80% of granulomatosis with polyangiitis.^1^^,^^2^ Despite optimal management, a significant proportion of patients progress to ESKD, requiring chronic dialysis or KT: 20% to 30% by 10 years.^3^^,^^4^

As in other kidney diseases, KT has emerged as the preferred treatment option for patients with AAV with ESKD, offering superior overall survival and quality of life compared with maintenance dialysis.^5^, ^6^, ^7^ Although relapses decrease after dialysis initiation,^8^, ^9^, ^10^ and further diminishes posttransplantation,^5^^,^^11^ they are associated with allograft loss.^12^ Overall and graft survivals appeared similar for patients with AAV when compared with other transplant recipients,^13^, ^14^, ^15^ though with some discrepancies between studies. In fact, most outcome data come from single-center reports or older cohorts,^11^^,^^13^^,^^14^ leaving uncertainty about long-term patient and graft survival in the era of modern immunosuppression and vasculitis therapies. Consequently, it remains unclear whether advances in vasculitis treatment and transplant care over the past decade have improved patient and graft outcomes after KT in AAV.

Despite the importance of optimal timing for KT in patients with AAV, current guidelines are limited. Based on a single survey-based study,^16^ suggesting poorer patient survival with early transplantation (< 12 months of remission), Kidney Disease: Improving Global Outcomes^17^ recommends a minimum of 6 months of complete clinical remission before considering KT. There is currently no French or European-specific recommendation on the management of these patients before or after transplantation.^18^^,^^19^ In fact, optimal timing for KT and factors associated with posttransplant outcomes (relapse, rejection, graft survival, and patient survival) have been poorly studied, mostly on small-scale studies^11^^,^^13^^,^^14^ (or macroscopic registry studies^20^^,^^21^), conducted in an earlier era, and with controversial results.

To address these critical knowledge gaps, we conducted an observational, retrospective, multicenter study, in the era of modern immunosuppressive regimens. We aimed to describe the incidence of DGF, graft failure, relapse, acute rejection, and death of patients with AAV-GN compared with a matched control group; and to investigate the risk factors associated with these events. We also analyzed the impact of various delays (including between ESKD and KT, and between waitlisting and KT) on these outcomes to identify the potentially optimal timeframe for KT.

## Methods

### Selection of Patients

This retrospective study included adult patients who received a kidney transplant in 12 French transplant centers (Spiesser Group), sharing a common transplant database (ASTRE).^22^, ^23^, ^24^ This database was used to identify patients transplanted between 2005 and 2023 for whom ESKD was caused by AAV-GN (cases) or other reasons (controls).

#### Selection of Cases

AAV diagnosis was based on the Chapel Hill Consensus Conference.^24^ AAV-GN diagnosis was based on active kidney involvement (active urinary sediment with hematuria, proteinuria and/or impaired kidney function) and, in most cases, confirmed by kidney biopsy showing pauci-immune GN. When kidney biopsy was not performed, ANCA positivity was mandatory. Some cases were included twice if they received > 1 transplant over time (*n* = 6).

#### Selection of Controls

Each AAV-GN case was systematically matched with 2 controls of the same center. Controls were blindly selected from the same database (ASTRE) among all patients transplanted during the study period (2005–2023) for ESKD because of any cause other than AAV-GN. The matching process prioritized the following criteria in hierarchical order: (i) same transplant center (mandatory), (ii) same sex when possible, (iii) recipient’s age within ± 5 years when possible, and (iv) transplant period within ± 5 years when possible (Supplementary Figure S1). When multiple potential controls met these criteria, selection was performed by minimizing differences of age and period as much as possible. This matching strategy ensured that controls were representative of the general kidney transplant population at each center during the study period.

### Data Collection

For all patients, cases or controls, pretransplant and posttransplant clinical, biological, histological, and treatment data (Table 1) were manually retrieved from individual medical records. Glomerular filtration rate was estimated by using the Chronic Kidney Disease Epidemiology Collaboration research group equation.^25^ Patients who returned to dialysis were assigned a creatinine value of 500 μmol/l and an estimated glomerular filtration rate of 5 ml/min. For cases, ANCA were assessed in indirect immunofluorescence (IIF) and/or quantitative enzyme immunoassay (EIA).

**Table 1:** Description of patients

Characteristics	*N*	Overall, *N* = 618	AAV-GN, *n* = 206	CTRL, *n* = 412	*P*-value
Baseline characteristics					
Male sex, *n* (%)	618	414 (67%)	137 (67%)	277 (67%)	0.9
BMI (kg/m^2^)	615	25.6 (4.5)	24.7 (4.1)	26.0 (4.6)	<0.001
Hypertension, *n* (%)	593	519 (88%)	174 (85%)	345 (89%)	0.2
Diabetes, *n* (%)	617	115 (19%)	23 (11%)	92 (22%)	<0.001
Kidney disease, *n* (%)	618				<0.001
AAV-GN		206 (33%)	206 (100%)	-	
ADPKD		80 (13%)	-	80 (19%)	
Immunological (not AAV)		75 (12%)	-	75 (18%)	
Diabetic		47 (7.6%)	-	47 (11%)	
Urologic		47 (7.6%)	-	47 (11%)	
Vascular		37 (6.0%)	-	37 (9.0%)	
Genetic (not ADPKD)		11 (1.8%)	-	11 (2.7%)	
Others		41 (6.6%)	-	41 (10.0%)	
Unknown		74 (12%)	-	74 (18%)	
Presentation at AAV-GN diagnosis					
Age (yrs)	200	-	52 (13)	-	
Kidney involvement, *n* (%)	203	-	203 (100%)	-	
Creatinine (μmol/l)	134	-	533 (364)	-	
Need for KRT within 30 d, *n* (%)	160	-	72 (45%)	-	
Proteinuria (g/g)	119	-	2.75 (2.77)	-	
Hematuria, *n* (%)	126	-	120 (95%)	-	
Kidney biopsy, *n* (%)	502	273 (54%)	172 (93%)	101 (32%)	<0.001
Lung involvement, *n* (%)	185	-	64 (35%)	-	
Heart involvement, *n* (%)	184	-	6 (3.3%)	-	
Neurological involvement, *n* (%)	184	-	13 (7.1%)	-	
ENT involvement, *n* (%)	185	-	39 (21%)	-	
Immunological findings					
Presence of ANCA, *n* (%)	193	-	189 (98%)	-	
Anti-PR3	177	-	49 (28%)	-	
Anti-MPO	176	-	128 (73%)	-	
Anti-GBM^a^	108	-	1 (0.9%)	-	
Therapeutic management of AAV-GN					
Induction remission therapy					
Plasma exchange, *n* (%)	185	-	64 (35%)	-	
Methylprednisolone pulses, *n* (%)	183	-	168 (92%)	-	
Prednisone, *n* (%)	195	-	183 (94%)	-	
Cyclophosphamide, *n* (%)	192	-	150 (78%)	-	
Rituximab, *n* (%)	191	-	19 (9.9%)	-	
Maintenance therapy					
Prednisone, *n* (%)	160	-	134 (84%)	-	
Cyclophosphamide, *n* (%)	177	-	19 (11%)	-	
Rituximab, *n* (%)	178	-	35 (20%)	-	
Azathioprine, *n* (%)	178	-	65 (37%)	-	
Mycophenolic acid, *n* (%)	178	-	30 (17%)	-	
Outcomes before KT					
AAV relapse	199	-	56 (28%)	-	
Preemptive transplantation	618	69 (11%)	15 (7.3%)	54 (13%)	0.030

AAV-GN, ANCA-associated vasculitis with glomerulonephritis; ADPKD, autosomal dominant polycystic kidney disease; ANCA, antineutrophil cytoplasmic autoantibody; BMI, body mass index; CTRL, controls; ENT, ear-nose-throat; GBM, glomerular basement membrane; KRT, kidney replacement therapy; KT, kidney transplantation; MPO, myeloperoxidase; PR3, proteinase 3; -, not applicable.

a One patient was found with anti-GBM antibodies at diagnosis, without sign of anti-GBM associated glomerulonephritis (pauci-immune glomerulonephritis on kidney biopsy).

### ANCA Status at the Time of KT

When available, ANCA status at the time of transplantation was collected within a timeframe of 3 months before KT to the day of KT. IIF and EIA status (positive or negative) at the time of transplantation were interpreted regardless of ANCA subtype (cANCA/pANCA, myeloperoxidase or proteinase 3), according to each center reference values. Only the positive or negative status was considered. Analyses were then conducted considering the following: (i) ANCA status according to ≥ 1 test (IIF or EIA): negative versus positive in ≥ 1 test, (ii) ANCA status according to IIF only: negative versus positive, (iii) ANCA status according to EIA only: negative versus positive, (iv) ANCA status according to both tests (IIF and EIA): double negative, double positive, or discordant (positive IIF only or positive EIA only) (Supplementary Figure S2).

### Definitions and Outcomes of Interest

Posttransplant outcomes were defined as follows:

1. DGF was defined as previously^26^: requirement for ≥ 1 dialysis session within 7 days of transplantation.
2. Kidney allograft failure (i.e., ESKD) was defined as the need of KRT for ≥ 3 months or the need for a new KT.
3. AAV relapse was defined as previously^27^: renal flares were diagnosed based on biopsy evidence of pauci-immune GN and clinical presence of hematuria, proteinuria and/or increase in serum creatinine, in the absence of other causes of renal allograft dysfunction. Extrarenal flare was diagnosed based on symptoms or radiological evidence of disease activity requiring an increase or a change in immunosuppression. ANCA positivity was not mandatory. In the control group, recurrence of initial nephropathy was not considered.
4. Acute rejection was defined as in Banff classification,^28^ acute antibody-mediated rejection and acute T-cell–medicated rejection were recorded but considered together for subsequent analyses. Borderline rejections were considered as part of acute T-cell–medicated rejection. Chronic rejection events were also recorded.

### Statistical Analysis

Continuous variables were described with mean ± SD, except for delays between events, presented as median (1st–3rd quartiles). Categorical variables were described with counts and percentages. Data were compared using the *t* test for continuous variables, and χ^2^ test (or Fisher exact test, if necessary) for categorical variables.

For the estimation of patient survival, Kaplan–Meier analyses were performed, and survival curves were compared with a log-rank test. The cumulative incidence of the above-mentioned outcomes of interest were evaluated with death as a competing event using the cumulative incidence competing risk method.^29^ Cumulative incidence curves were compared using Gray’s test.

Cox regression analyses were used to do the following: (i) adjust event-free survival when comparing cases and controls, and (ii) perform multivariable analyses to examine factors associated with outcomes in the AAV-GN group. Multivariate Cox regression analysis included all parameters with *P* < 0.1 in the univariable analysis. To simplify the multivariable models, the number of variables was limited using manual step-by-step backward selection with a removal criterion of *P* > 0.1. HR with 95% confidence intervals are reported. A logistic regression analysis was performed to examine factors associated with DGF in the AAV-GN group. Multivariable analysis was carried out as described above. Odds ratios with 95% confidence intervals are reported. In multivariable models, variance inflation factor was computed to check the absence of collinearity against dependent variables. No imputation of missing data was performed. Statistical analyses were performed using R v4.0. All tests were 2-sided, and a *P*-value < 0.05 was considered statistically significant.

### Ethical Issues

Our cohort has been declared and authorized by the “Commission Nationale Informatique et Libertés” (agreement number 202200131/ar23-0077v0). This study was approved by the local ethics committee of Angers University Hospital (2022-175). Because the nature of the study and in accordance with French law, the patients’ written consent was not mandatory. The clinical and research activities being reported are consistent with the Principles of the Declaration of Istanbul as outlined in the “Declaration of Istanbul on Organ Trafficking and Transplant Tourism.”

## Results

### Description of AAV-GN Cases

A total of 206 patients received a KT for ESKD secondary to AAV-GN. Cases characteristics are summarized in Table 1, Table 2. Patients with AAV-GN were diagnosed between 1980 and 2020. The mean age at diagnosis was 52 ± 13 years, two-thirds (67%) of patients were males, and mostly (72%) found with myeloperoxidase-ANCA at diagnosis. Before KT, 56 (28%) experienced at least 1 relapse. The mean age at KT was 59 ± 11 years and patients were transplanted after a median waiting time of 14 (7–23) months. Fifteen patients (7.3%) were transplanted preemptively, and the median time in dialysis before KT was 30 (18–46) months for the other patients (Supplementary Figure S3). Donor source was mostly deceased donors (90%). There was no ABO incompatible transplantation and the mean calculated panel reactive antibody was 18 ± 31%. At the time of transplantation, ANCA status (IIF or EIA) was evaluated in 167 (80%) patients. It was positive in ≥ 1 technique for 99 (59%). Only 97 patients (47%) were tested using both techniques (IIF and EIA), 37 patients (38%) being positive in both (Supplementary Figure S2). ANCA positive patients less frequently (91% vs. 99%) received steroids as KT maintenance regimen, but no other major difference was found when compared with ANCA negative patients (Supplementary Table S1). Median follow-up after KT was 52 (26–84) months.

**Table 2:** Kidney transplantation procedure and outcomes

Characteristics	*N*	Overall, *N* = 618	AAV-GN, *n* = 206	CTRL, *n* = 412	*P*-value
Status at KT					
Recipient age (yrs)	618	58 (12)	59 (11)	58 (12)	0.071
First transplantation	618	551 (89%)	190 (92%)	361 (88%)	0.082
Calculated PRA	459	19 (31)	18 (31)	19 (32)	0.6
ANCA status at KT					
According to IIF only - positive, *n* (%)	143	-	76 (53%)	-	
According to EIA only - positive, *n* (%)	120	-	59 (49%)	-	
According to ≥ 1 test (IIF or EIA)	167				
Negative, *n* (%)		-	68 (41%)	-	
≥ 1 positive, *n* (%)		-	99 (59%)	-	
According to both tests (IIF and EIA)	97				
Double negative, *n* (%)		-	35 (36%)	-	
Discordant, *n* (%)		-	25 (26%)	-	
IIF positive & EIA negative, *n* (%)		-	21 (22%)	-	
IIF negative & EIA positive, *n* (%)		-	4 (4.1%)	-	
Double positive, *n* (%)		-	37 (38%)	-	
Transplantation procedure					
Donor age (yrs)	591	59 (15)	59 (14)	59 (15)	0.4
Deceased donor, *n* (%)	618	571 (92%)	186 (90%)	385 (93%)	0.2
ABO incompatibility, *n* (%)	534	1 (0.2%)	0 (0%)	1 (0.3%)	>0.9
Donor creatinine (μmol/l)	541	79 (42)	81 (48)	78 (39)	0.5
Cold ischemia time (h)	606	14 (7)	14 (7)	15 (7)	0.078
HLA-mismatches (total)	618	4.56 (1.70)	4.57 (1.64)	4.56 (1.73)	>0.9
HLA-A	612	1.25 (0.68)	1.27 (0.65)	1.24 (0.69)	0.7
HLA-B	612	1.49 (0.62)	1.43 (0.60)	1.52 (0.63)	0.10
HLA-DR	612	1.06 (0.69)	1.10 (0.67)	1.03 (0.69)	0.3
HLA-DQ	530	0.94 (0.74)	0.91 (0.71)	0.95 (0.75)	0.5
Immunosuppressive regimen					
Induction therapy					
Anti-thymocyte globulin, *n* (%)	614	198 (32%)	71 (34%)	127 (31%)	0.4
Basiliximab, *n* (%)	614	406 (66%)	133 (65%)	273 (67%)	0.6
Prednisone, *n* (%)	613	595 (97%)	197 (97%)	398 (97%)	0.6
Maintenance regimen					
Calcineurin inhibitors, *n* (%)	617	613 (99%)	206 (100%)	407 (99%)	0.3
Mycophenolic acid, *n* (%)	615	593 (96%)	193 (94%)	400 (98%)	0.010
Prednisone, *n* (%)	598	485 (81%)	188 (94%)	297 (75%)	<0.001
mTOR inhibitors, *n* (%)	609	106 (17%)	50 (25%)	56 (14%)	<0.001
Azathioprine, *n* (%)	609	29 (4.8%)	16 (7.9%)	13 (3.2%)	0.011
Outcomes after KT					
Follow-up duration (mo)	618	69 (52)	52 (26, 84)	60 (30, 104)	0.021
DGF, *n* (%)	614	114 (19%)	37 (18%)	77 (19%)	0.8
Allograft failure, *n* (%)	618	79 (13%)	30 (15%)	49 (12%)	0.3
Relapses					
All, *n* (%)	206	15 (7.3%)	15 (7.3%)	-	-
With kidney involvement, *n* (%)	206	12 (5.8%)	12 (5.8%)	-	-
Rejection, *n* (%)	609	114 (19%)	39 (19%)	75 (19%)	0.9
Acute rejection (AR), *n* (%)	608	108 (18%)	38 (19%)	70 (17%)	0.7
TCMR, *n* (%)	599	70 (12%)	22 (11%)	48 (12%)	0.6
ABMR, *n* (%)	600	25 (4.2%)	11 (5.4%)	14 (3.5%)	0.3
Mixed AR, *n* (%)	616	12 (1.9%)	5 (2.4%)	7 (1.7%)	0.5
Chronic rejection	601	23 (3.8%)	8 (3.9%)	15 (3.8%)	>0.9
Death	617	131 (21%)	50 (24%)	81 (20%)	0.2

-, not applicable; AAV-GN, ANCA-associated vasculitis with glomerulonephritis; ABMR, antibody-mediated rejection; ANCA, antineutrophil cytoplasmic autoantibody; AR, acute rejection; CTRL, controls ; DGF, delayed graft function; EIA, enzyme immunoassays; GBM, glomerular basement membrane; HLA, human leukocyte antigen; IIF, indirect immunofluorescence; KRT, kidney replacement therapy; KT, kidney transplantation; mTOR, mammalian target of rapamycin; PRA, panel reactive antibody; TCMR, T-cell–mediated rejection.

### Description of Control Patients

Characteristics of the 412 controls included in the study are summarized in Table 1, Table 2. Overall, 100%, 95%, 89%, and 80% of cases were well-matched with controls regarding transplant center, year of transplantation, sex, and recipient age, respectively (Supplementary Figure S2).

Mean age at KT was 58 ± 12 years. Fifty-five patients (13%) were transplanted preemptively, and the median time in dialysis before KT was 25([9–47) months for the other patients (Supplementary Figure S3). Donor source was mostly deceased donors (94%). There was 1 case of ABO incompatibility and mean calculated panel reactive antibody was 19 ± 32%. Causes of ESKD were mostly led by autosomal dominant polycystic kidney disease (19%), immunological diseases (18%), and unknown causes (18 %). Median follow-up after KT was 60 (30–104) months.

### Kidney Function and Survival

#### DGF and Kidney Function Evolution

There was no significant difference in the incidence of DGF between groups, involving 37 (18%) and 77 (19%) patients in the AAV-GN and control group, respectively (*P* = 0.9, Table 1). Factors associated with the occurrence of DGF, in the AAV-GN group, in univariable analysis are described in Supplementary Table S2. ANCA positivity at the time of KT was not associated with the occurrence of DGF. In multivariable analysis, cold ischemia time (odds ratio = 1.07 [1.00–1.14], *P* = 0.043), previous AAV relapse before KT (odds ratio = 0.23 (0.05–0.70), *P* = 0.021) and human leukocyte antigen–DQ mismatches (odds ratio = 0.51 [0.28–0.91], *P* = 0.027) were associated with the occurrence of DGF (Supplementary Table S2).

**Figure 1:**
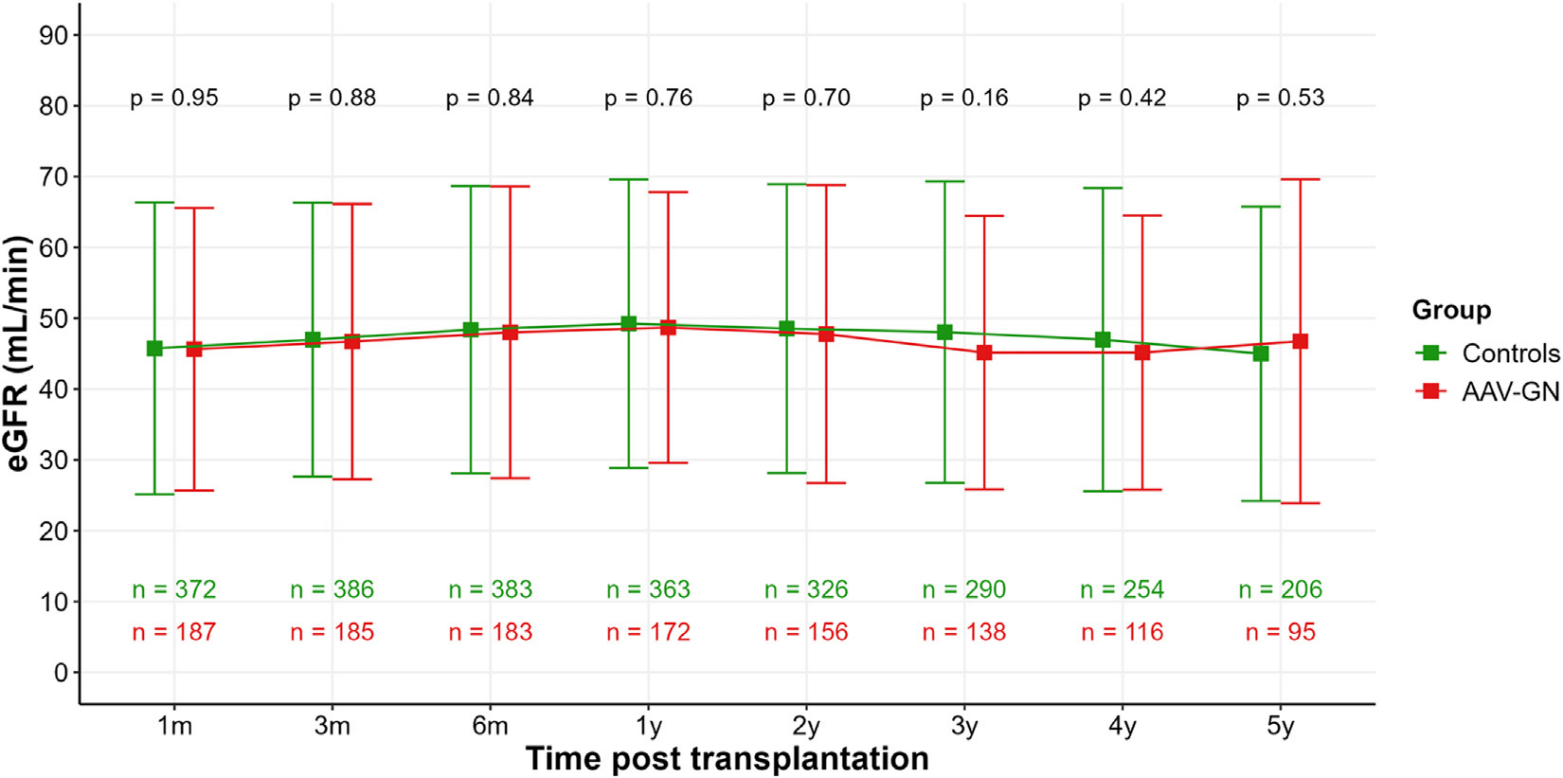
Estimated glomerular filtration rate (eGFR) evolution after kidney transplantation. Data are represented as mean ± SD. AAV-GN, ANCA-associated vasculitis with glomerulonephritis; ANCA, antineutrophil cytoplasmic autoantibody; eGFR, estimated glomerular filtration rate.

There was no significant difference in kidney function evolution overtime; creatinine levels were 148 ± 68 and 146 ± 76 μmol/l at 6 months and 1 year, respectively; and estimated glomerular filtration rate levels were 49 ± 20 and 49 ± 20 ml/min at 6 months and 1 year, in the whole cohort (including patients back on chronic dialysis). Similar values were observed over the first 5 years of follow-up (Figure 1).

#### Graft Survival

**Figure 2:**
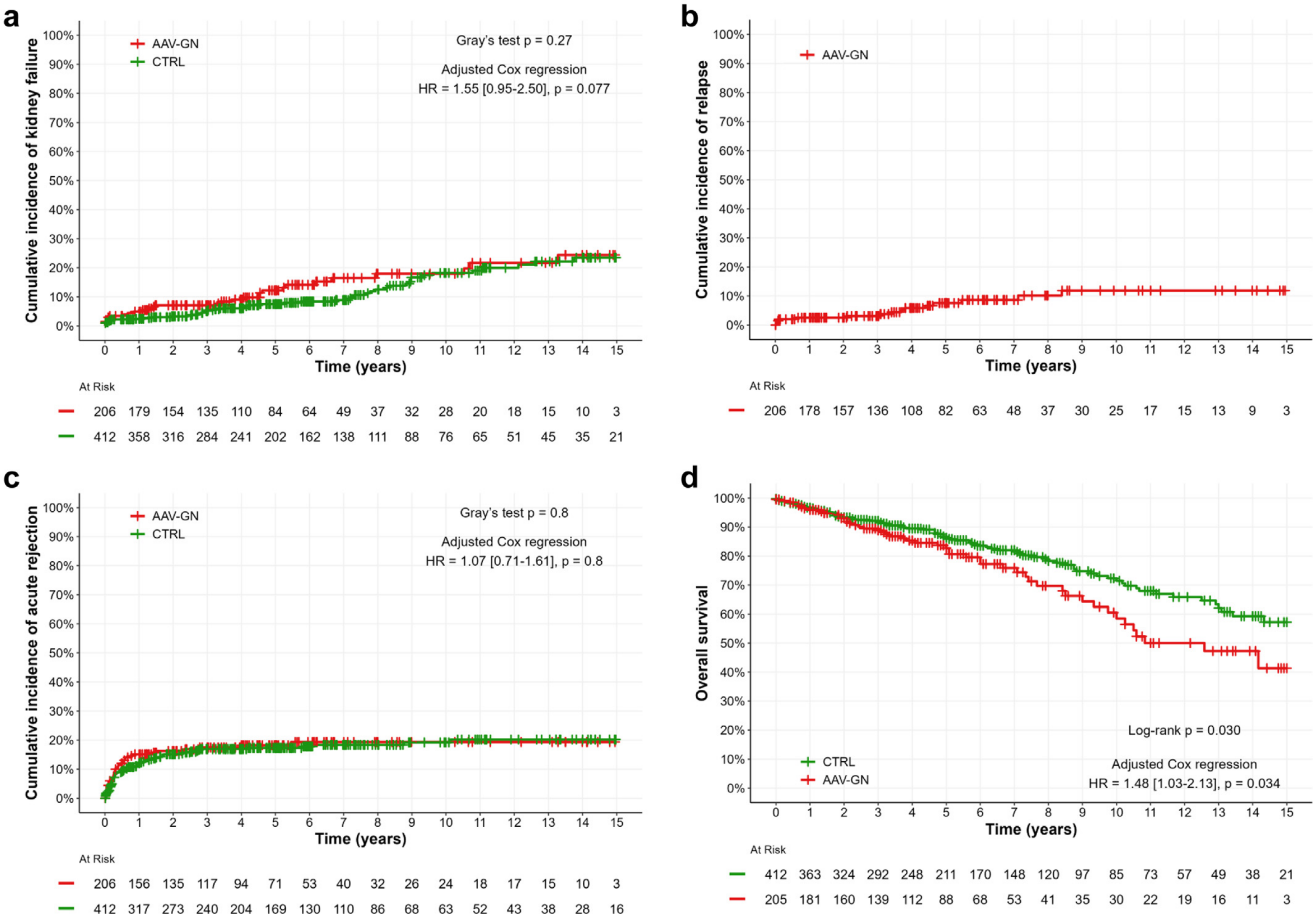
Event-free survival after kidney transplantation. Cumulative incidence of (a) graft failure, (b) relapses or (c) acute rejection, and (d) overall survival. AAV-GN, ANCA-associated vasculitis with glomerulonephritis.

A total of 79 graft failures occurred during follow-up: 30 (15%) and 49 (12%) in AAV-GN and control group, respectively. In univariable analysis, there was no significant difference in the cumulative incidence of graft failure when comparing the AAV-GN group with the control group (4.9% vs. 2.5% at 1 year, 12% vs. 7.5% at 5 years, 18% vs. 18% at 10 years, *P* = 0. 27, Figure 2a). However, in multivariable analysis, patients with AAV-GN were at higher risk of graft failure, though it did not reach statistical significance (HR = 1.55 [0.95–2.50], *P* = 0.077, Supplementary Table S3).

In the AAV-GN group, in multivariable analysis, recipient age at KT (HR = 0.93 [0.88–0.98] /1-yr increment, *P* = 0.005), donor age (HR = 1.11 [1.06–1.17]/1-yr increment, *P* < 0.001), steroid (HR: 0.18 [0.05–0.62], *P* = 0.007) and AZA (HR = 3.73 [1.44–9.69], *P* = 0.007) used as maintenance therapy, DGF (HR = 3.43 [1.44–8.19], *P* = 0.005), posttransplant relapse (HR = 3.49 [1.38–8.82], *P* = 0.008) or any type of rejection (HR = 3.87 [1.65-9.08, *P* = 0.002) were associated with graft survival (Table 3). Of note, there was no difference in graft survival regarding ANCA status at KT (Table 3 and Supplementary Figure S4A).

**Table 3:** Factors associated with graft failure (in the AAV-GN cohort)

Characteristics	Univariable					Multivariable (simplified)				
	*n*	Event *n*	HR	95% CI	*P*-value	*n*	Event *n*	HR	95% CI	*P*-value
Baseline characteristics										
Male sex (vs. female)	206	30	2.00	0.82–4.91	0.13					
BMI (kg/m^2^)	206	30	0.96	0.88–1.05	0.4					
Hypertension (vs. no)	204	30	0.46	0.21–1.00	0.051					
Diabetes (vs. no)	206	30	1.17	0.35–3.88	0.8					
Presentation at AAV-GN diagnosis										
Age (yrs)	200	29	1.03	1.00–1.07	0.064					
Kidney involvement (vs. no)	206	30	Inf.	Inf.	-					
Creatinine (μmol/l)	133	21	1	1.00–1.00	>0.9					
Need for KRT within 30 d (vs. no)	160	22	0.94	0.40–2.18	0.9					
Proteinuria (g/g)	119	16	1.11	0.98–1.25	0.089					
Hematuria (vs. no)	126	17	0.39	0.09–1.73	0.2					
Lung involvement (vs. no)	185	27	1.22	0.57–2.63	0.6					
Heart involvement (vs. no)	184	27	1.02	0.14–7.53	>0.9					
Neurological involvement (vs. no)	184	27	1.27	0.30–5.39	0.7					
ENT involvement (vs. no)	185	27	0.91	0.36–2.26	0.8					
Immunological findings										
Presence of ANCA (vs. no)	193	27	0.23	0.03–1.76	0.2					
Anti-PR3 (vs. anti-MPO)	176	26	0.65	0.26–1.61	0.3					
Therapeutic management of AAV-GN										
Induction remission therapy										
Plasma exchange (vs. no)	185	27	2.04	0.95–4.37	0.067					
Methylprednisolone pulses (vs. no)	183	27	0.73	0.22–2.43	0.6					
Prednisone (vs. no)	195	28	0.37	0.11–1.25	0.11					
Cyclophosphamide (vs. no)	192	28	0.72	0.31–1.71	0.5					
Rituximab (vs. no)	191	28	1.21	0.28–5.22	0.8					
Maintenance therapy										
Prednisone (vs. no)	160	23	0.42	0.16–1.07	0.068					
Cyclophosphamide (vs. no)	177	24	1.06	0.31–3.55	>0.9					
Rituximab (vs. no)	178	24	2.32	0.89–6.03	0.086					
Azathioprine (vs no)	178	24	0.78	0.33–1.83	0.6					
Mycophenolic acid (vs. no)	178	24	0.81	0.28–2.39	0.7					
Outcomes before KT										
Relapse	199	29	0.98	0.43–2.22	>0.9					
Preemptive transplantation	206	30	0.42	0.06–3.11	0.4					
Status at KT										
Recipient age (yrs)	206	30	1.02	0.99–1.07	0.2	187	29	0.93	0.88–0.98	0.005
First transplantation	206	30	0.82	0.25–2.72	0.8					
Calculated PRA	157	18	0.99	0.97–1.01	0.4					
ANCA status at KT										
According to IIF only - positive	143	22	1.08	0.46–2.56	0.9					
According to EIA only - positive	120	21	1.18	0.50–2.78	0.7					
According to ≥ 1 test (IIF or EIA) - ≥ 1 positive	167	27	1.01	0.46–2.23	>0.9					
According to both tests (IIF and EIA)	97	16								
Double negative			Ref.	-	-					
Discordant			0.95	0.25–3.57	>0.9					
IIF positive & EIA negative			0.79	0.19–3.34	0.7					
IIF negative & EIA positive			2.35	0.27–20.2	0.4					
Double positive			1.21	0.38–3.84	0.7					
Transplantation procedure										
Donor age (yrs)	195	29	1.05	1.02–1.09	0.002	187	29	1.11	1.06–1.17	<0.001
Deceased donor (vs no)	206	30	2.15	0.29–15.9	0.5					
Donor creatinine (per 50 μmol/l increment)	180	24	0.95	0.61–1.48	0.8					
Cold ischemia time (h)	205	30	1.02	0.97–1.08	0.4					
HLA-mismatches (total)	205	30	0.97	0.78–1.22	0.8					
HLA-A	205	30	0.79	0.46–1.35	0.4					
HLA-B	205	30	1.17	0.62–2.18	0.6					
HLA-DR	205	30	1.08	0.63–1.88	0.8					
HLA-DQ	180	27	0.8	0.47–1.36	0.4					
Immunosuppressive regimen										
Induction therapy										
Anti-thymocyte globulin (vs. no)	206	30	0.64	0.29–1.45	0.3					
Basiliximab (vs. no)	206	30	1.21	0.57–2.59	0.6					
Prednisone (vs. no)	204	29	1.03	0.14–7.61	>0.9					
Maintenance regimen										
Calcineurin inhibitors (vs. no)	206	30	Inf.	Inf.	-					
Mycophenolic acid (vs. no)	206	30	0.65	0.20–2.13	0.5					
Prednisone (vs. no)	201	30	0.34	0.12–0.97	0.044	187	29	0.18	0.05–0.62	0.007
mTOR inhibitors (vs. no)	201	30	0.84	0.36–1.97	0.7					
Azathioprine (vs. no)	203	30	4.09	1.72–9.75	0.001	187	29	3.73	1.44–9.69	0.007
Outcomes after KT										
DGF (vs. no)	204	30	2.47	1.15–5.29	0.02	187	29	3.43	1.44–8.19	0.005
Relapses										
All (vs. no)^a^	206	30	3.74	1.65–8.48	0.002	187	29	3.49	1.38–8.82	0.008
With kidney involvement (vs no)^b^	206	30	4.14	1.77–9.71	0.001					
Rejection (vs no)	204	30	4.38	2.13–8.99	<0.001	187	29	3.87	1.65–9.08	0.002
AR (vs. no)	204	30	4.11	2.01–8.43	<0.001					
TCMR (vs. no)	205	30	0.98	0.34–2.82	>0.9					
ABMR (vs. no)	205	30	8.29	3.66–18.8	<0.001					
Mixed AR (vs. no)	205	30	6.84	2.03–23.1	0.002					
Chronic rejection	205	30	3.4	1.29–9.01	0.014					

-, not applicable; AAV-GN, ANCA-associated vasculitis with glomerulonephritis; ABMR, antibody-mediated rejection; ADPKD, autosomal dominant polycystic kidney disease; ANCA, antineutrophil cytoplasmic autoantibody; AR, acute rejection; BMI, body mass index; CI, confidence interval; DGF, delayed graft function; EIA, enzyme immunoassays; HLA, human leukocyte antigen; HR, hazard ratio; IIF, indirect immunofluorescence; KRT, kidney replacement therapy; KT, kidney transplantation; MPO, myeloperoxidase; mTOR, mammalian target of rapamycin; PR3, proteinase 3; PRA, panel reactive antibody; TCMR, T-cell–mediated rejection.

When there was not enough data in some subgroups, Cox regression model could not converge (result is given as “Inf.”).

a One patient experienced a relapse (not involving the transplant) after graft failure (see Supplementary Table S4). Hence, for this analysis, this patient was considered as a non-relapser.

b An alternative multivariable model was built by replacing “any type of relapse” by “renal relapse”. Renal relapses were strongly associated with graft survival (HR = 4.72 [2.05–10.8], *P* < 0.001).

### Relapses

#### Description of Relapses

A total of 15 (patients 7.3%) with AAV-GN relapsed after transplantation. The median time between transplantation to relapse was 41 (7–55) months, ranging from 8 days to 8.4 years. Most relapsing patients were ANCA positive (in ≥ 1 assay) at the time of KT (11/13, 85%) and all were ANCA positive (in ≥ 1 assay) at the time of relapse (13/13, 100%). Characteristics of relapsing patients are described in Supplementary Table S4. Individual relapsing cases (presentation, management, outcomes) are described in Supplementary Table S5 and Supplementary Figure S5.

Patients experiencing relapses were more likely to experience graft failure when compared with patients who did not relapse (60% vs. 11%, *P* < 0.001, Supplementary Table S4). Indeed, relapses were strongly associated with decreased kidney survival (*P* < 0.001, Supplementary Figure S5A), including in multivariable analysis (HR = 3.49 [1.38–8.82], *P* = 0.008, Table 3). They were also more likely to experience acute rejection (40% vs. 17%, *P* = 0.039, Supplementary Table S4). Relapses were not associated with death (Table 6 and Supplementary Figure S6B). Similar results were found when considering renal relapses only; it was strongly associated with decreased kidney survival (*P* < 0.001, Supplementary Figure S6C), including in multivariable analysis (HR = 4.72 [2.05–10.8], *P* < 0.001, Table 3). Renal relapses were not associated with death (Table 6 and Supplementary Figure S6D).

**Table 6:** Factors associated with death (in the AAV-GN cohort)

Characteristics	Univariable					Multivariable (simplified)				
	*n*	Event *n*	HR	95% CI	*P*-value	*n*	Event *n*	HR	95% CI	*P*-value
Baseline characteristics										
Male sex (vs female)	205	50	1.11	0.61–2.04	0.7					
BMI (kg/m^2^)	205	50	0.99	0.93–1.06	0.8					
Hypertension (vs. no)	203	49	1.41	0.60–3.33	0.4					
Diabetes (vs. no)	205	50	1.74	0.74–4.13	0.2					
Presentation at AAV-GN diagnosis										
Age (yrs)	199	49	1.05	1.02–1.09	<0.001					
Kidney involvement (vs. no)	205	50	Inf.	Inf.	-					
Creatinine (μmol/l)	132	35	1.00	1.00–1.00	0.7					
Need for KRT within 30 d (vs. no)	159	41	0.87	0.47–1.61	0.6					
Proteinuria (g/g)	118	32	0.99	0.85–1.14	0.8					
Hematuria (vs. no)	125	31	0.32	0.11–0.91	0.033					
Lung involvement (vs. no)	184	47	0.83	0.44–1.56	0.6					
Heart involvement (vs. no)	183	47	0.61	0.08–4.46	0.6					
Neurological involvement (vs. no)	183	47	0.75	0.18–3.09	0.7					
ENT involvement (vs. no)	184	47	0.72	0.35–1.50	0.4					
Immunological findings										
Presence of ANCA (vs. no)	192	49	0.13	0.03–0.57	0.006					
Anti-PR3 (vs anti-MPO)	175	44	0.39	0.17–0.87	0.022	175	44	0.43	0.19–0.96	0.040
Therapeutic management of AAV-GN										
Induction remission therapy										
Plasma exchange (vs. no)	184	47	1.18	0.64–2.16	0.6					
Methylprednisolone pulses (vs. no)	182	46	1.38	0.43–4.46	0.6					
Prednisone (vs. no)	194	48	0.70	0.21–2.26	0.5					
Cyclophosphamide (vs. no)	191	47	1.14	0.53–2.46	0.7					
Rituximab (vs. no)	190	47	1.96	0.68–5.67	0.2					
Maintenance therapy										
Prednisone (vs. no)	159	38	0.59	0.27–1.29	0.2					
Cyclophosphamide (vs. no)	176	44	1.19	0.50–2.82	0.7					
Rituximab (vs. no)	177	44	1.60	0.69–3.72	0.3					
Azathioprine (vs. no)	177	43	0.71	0.37–1.37	0.3					
Mycophenolic acid (vs. no)	177	44	0.85	0.40–1.85	0.7					
Outcomes before KT										
Relapse	198	50	0.78	0.41–1.50	0.5					
Preemptive transplantation	205	50	1.48	0.58–3.74	0.4					
Status at KT										
Recipient age (yrs)	205	50	1.06	1.02–1.09	0.002	175	44	1.06	1.02–1.11	0.004
First transplantation	205	50	1.58	0.49–5.08	0.4					
Calculated PRA	156	32	1.01	1.00–1.02	0.3					
ANCA status at KT										
According to IIF only - positive	142	33	0.68	0.34–1.37	0.3					
According to EIA only - positive	120	25	0.92	0.42–2.02	0.8					
According to ≥ 1 test (IIF or EIA) - ≥ 1 positive	166	39	0.91	0.47–1.75	0.8					
According to both tests (IIF and EIA)	97	19								
Double negative			Ref.	-	-					
Discordant			1.03	0.36–2.93	>0.9					
IIF positive & EIA negative			0.93	0.30–2.83	0.9					
IIF negative & EIA positive			1.59	0.32–7.93	0.6					
Double positive			0.42	0.12–1.47	0.2					
Transplantation procedure										
Donor age (yrs)	194	48	1.02	1.00–1.04	0.080					
Deceased donor (vs. no)	205	50	0.66	0.24–1.87	0.4					
Donor creatinine (per 50 μmol/l increment)	179	45	0.99	0.72–1.36	>0.9					
Cold ischemia time (h)	204	50	1.00	0.95–1.04	0.9					
HLA-mismatches (total)	205	50	1.02	0.85–1.22	0.8					
HLA-A	204	50	1.10	0.71–1.69	0.7					
HLA-B	204	50	0.67	0.42–1.06	0.084					
HLA-DR	204	50	1.07	0.69–1.67	0.7					
HLA-DQ	179	43	1.20	0.80–1.79	0.4					
Immunosuppressive regimen										
Induction therapy										
Anti-thymocyte globulin (vs. no)	205	50	1.10	0.62–1.93	0.8					
Basiliximab (vs. no)	205	50	1.07	0.60–1.90	0.8					
Prednisone (vs. no)	203	49	0.79	0.19–3.24	0.7					
Maintenance regimen										
Calcineurin inhibitors (vs. no)	205	50	Inf.	Inf.	-					
Mycophenolic acid (vs. no)	205	50	1.66	0.40–6.83	0.5					
Prednisone (vs. no)	200	47	1.07	0.26–4.42	>0.9					
mTOR inhibitors (vs. no)	201	48	1.07	0.58–1.99	0.8					
Azathioprine (vs. no)	202	48	1.37	0.54–3.48	0.5					
Outcomes after KT										
DGF (vs. no)	203	48	0.92	0.43–1.97	0.8					
Allograft failure (vs. no)	205	50	0.82	0.35–1.94	0.7					
Relapses										
All (vs. no)	205	50	0.94	0.37–2.38	0.9					
With kidney involvement (vs. no)	205	50	1.06	0.38–2.97	>0.9					
Rejection (vs. no)	203	49	1.24	0.67–2.32	0.5					
AR (vs. no)	203	50	1.32	0.71–2.45	0.4					
TCMR (vs. no)	204	50	1.15	0.55–2.38	0.7					
ABMR (vs. no)	204	50	1.59	0.57–4.43	0.4					
Mixed AR (vs. no)	204	50	1.44	0.20–10.6	0.7					
Chronic rejection	204	50	1.26	0.45–3.53	0.7					

-, means not applicable; AAV-GN, ANCA-associated vasculitis with glomerulonephritis; ABMR, antibody-mediated rejection; ADPKD, autosomal dominant polycystic kidney disease; ANCA, antineutrophil cytoplasmic autoantibody; AR, acute rejection; BMI, body mass index; CI, confidence interval; DGF, delayed graft function; EIA, enzyme immunoassays; HR, hazard ratio; IIF, indirect immunofluorescence; KRT, kidney replacement therapy; KT, kidney transplantation; mTOR, mammalian target of rapamycin; PR3, proteinase 3; PRA, panel reactive antibody; TCMR, T-cell–mediated rejection.

When there was not enough data in some subgroups, Cox regression model could not converge (result is given as "Inf.").

#### Relapse-Free Survival

Cumulative incidence of relapse was 2.5%, 7.6%, and 12% at 1, 5, and 10 years, respectively, in the AAV-GN group (Figure 2b). In multivariable analysis, only the presence of ANCA at AAV-GN diagnosis (HR = 0.04 [0.01–0.24], *P* < 0.001) (Table 4). ANCA status tended to be associated with relapse-free survival, but with discordant results according to the type of test used as follows: (i) when considering the maximum number of subjects with available testing (IIF or EIA, *n* = 158), ≥ 1 positive ANCA test tended to be associated (HR = 4.17 [0.91–19.0], *P* = 0.065); (ii) when considering IIF only (*n* = 134), similar results were found (HR = 3.81 [0.80–18.2], *P* = 0.094); (iii) when considering EIA only (*n* = 115), no association were found; and (iv) when considering patients with both IIF and EIA testing (*n* = 92), no association was found, whether considering discordant or simple positive (vs. negative) or double positive (vs. negative) (Supplementary Table S6, Supplementary Figure S4B). The analysis was, however, limited because of a small number of events. The effect of ANCA positivity was not influenced by the initial subtype of ANCA either (myeloperoxidase or proteinase 3) (Supplementary Figure S6).

**Table 4:** Factors associated with relapses (in the AAV-GN cohort)

Characteristics	Univariable					Multivariable (simplified)				
	*n*	Event *n*	HR	95% CI	*P*-value	*N*	Event *n*	HR	95% CI	*P*-value
Baseline characteristics										
Male sex (vs. female)	206	15	3.42	0.77–15.1	0.11					
BMI (kg/m^2^)	206	15	1.05	0.94–1.17	0.4					
Hypertension (vs. no)	204	15	0.51	0.16–1.61	0.3					
Diabetes (vs. no)	206	15	1.56	0.35–6.95	0.6					
Presentation at AAV-GN diagnosis										
Age (yrs)	200	14	1.00	0.96–1.05	0.9					
Kidney involvement (vs. no)	206	15	Inf.	Inf.	-					
Creatinine (μmol/l)	133	12	1.00	1.00–1.00	0.6					
Need for KRT within 30 d (vs. no)	160	12	1.18	0.38–3.66	0.8					
Proteinuria (g/g)	119	8	1.09	0.90–1.31	0.4					
Hematuria (vs. no)	126	10	0.45	0.06–3.59	0.5					
Lung involvement (vs. no)	185	13	1.11	0.36–3.41	0.8					
Heart involvement (vs. no)	184	13	Inf.	Inf.	-					
Neurological involvement (vs. no)	184	13	3.25	0.72–14.7	0.13					
ENT involvement (vs. no)	185	14	1.48	0.46–4.73	0.5					
Immunological findings										
Presence of ANCA (vs. no)	193	15	0.05	0.01–0.25	<0.001	158	13	0.05	0.01–0.31	0.001
Anti-PR3 (vs. anti-MPO)	176	14	0.62	0.17–2.22	0.5					
Therapeutic management of AAV-GN										
Induction remission therapy										
Plasma exchange (vs. no)	185	15	0.99	0.34–2.89	>0.9					
Methylprednisolone pulses (vs. no)	183	15	0.34	0.10–1.22	0.10					
Prednisone (vs. no)	195	15	0.23	0.05–1.08	0.063					
Cyclophosphamide (vs. no)	192	15	0.61	0.19–1.93	0.4					
Rituximab (vs. no)	191	15	1.02	0.13–7.91	>0.9					
Maintenance therapy										
Prednisone (vs. no)	160	13	0.49	0.14–1.81	0.3					
Cyclophosphamide (vs. no)	177	14	2.43	0.67–8.82	0.2					
Rituximab (vs. no)	178	14	0.47	0.06–3.61	0.5					
Azathioprine (vs. no)	178	14	0.84	0.28–2.51	0.8					
Mycophenolic acid (vs. no)	178	14	1.01	0.28–3.64	>0.9					
Outcomes before KT										
Relapse	199	15	1.30	0.44–3.80	0.6					
Preemptive transplantation	206	15	0.87	0.11–6.64	0.9					
Status at KT										
Recipient age (yrs)	206	15	1.00	0.95–1.05	>0.9					
First transplantation	206	15	Inf.	Inf.	-					
Calculated PRA	157	7	0.00	0.00–Inf	>0.9					
ANCA status at KT										
According to IIF only - positive	143	10	3.58	0.76–16.9	0.11					
According to EIA only - positive	120	12	1.08	0.35–3.34	0.9					
According to ≥ 1 test (IIF or EIA) - ≥ 1 positive	167	13	3.77	0.84–17.0	0.084	158	13	4.17	0.91–19.0	0.065
According to both tests (IIF and EIA)										
Double negative			Ref.	-	-					
Discordant			2.5	0.46–13.7	0.3					
IIF positive & EIA negative			3.08	0.56–16.9	0.2					
IIF negative & EIA positive			0	0.00–Inf	>0.9					
Double positive			1.37	0.23–8.25	0.7					
Transplantation procedure										
Donor age (yrs)	195	14	0.99	0.96–1.03	0.8					
Deceased donor (vs. no)	206	15	Inf.	Inf.	-					
Donor creatinine (per 50 μmol/l increment)	180	14	0.79	0.39–1.62	0.5					
Cold ischemia time (h)	205	15	0.98	0.91–1.06	0.7					
HLA-mismatches (total)	206	15	1.09	0.79–1.52	0.6					
HLA-A	205	15	0.92	0.42–2.02	0.8					
HLA-B	205	15	1.10	0.46–2.64	0.8					
HLA-DR	205	15	1.06	0.49–2.30	0.9					
HLA-DQ	180	15	1.10	0.56–2.18	0.8					
Immunosuppressive regimen										
Induction therapy										
Anti-thymocyte globulin (vs. no)	206	15	0.41	0.12–1.46	0.2					
Basiliximab (vs. no)	206	15	1.79	0.57–5.63	0.3					
Prednisone (vs. no)	204	14	0.48	0.06–3.70	0.5					
Maintenance regimen										
Calcineurin inhibitors (vs. no)	206	15	Inf.	Inf.	-					
Mycophenolic acid (vs no)	206	15	0.47	0.11–2.10	0.3					
Prednisone (vs. no)^a^	201	15	0.79	0.10–6.02	0.8					
mTOR inhibitors (vs. no)	201	15	Inf.	Inf.	-					
Azathioprine (vs. no)	203	15	1.53	0.34–6.80	0.6					
Outcomes after KT										
DGF (vs. no)	204	15	0.75	0.17–3.31	0.7					

-, means not applicable. AAV-GN, ANCA-associated vasculitis with glomerulonephritis; ABMR, antibody-mediated rejection; ADPKD, autosomal dominant polycystic kidney disease; ANCA, antineutrophil cytoplasmic autoantibody; AR, acute rejection; BMI, body mass index; CI, confidence interval; DGF, delayed graft function; EIA, enzyme immunoassays; HR, hazard ratio; IIF, indirect immunofluorescence; KRT, kidney replacement therapy; KT, kidney transplantation; MPO, myeloperoxidase; mTOR, mammalian target of rapamycin; PR3, proteinase 3; PRA, panel reactive antibody; TCMR, T-cell–mediated rejection.

When there was not enough data in some subgroups, Cox regression model could not converge (result is given as "Inf.").

a Because KT maintenance regimen with steroids was differentially distributed between ANCA positive and negative patient, it was forced into an alternative multivariable model. Maintenance with steroid was not associated with relapses (HR = 0.77 [0.10–6.16], *P* = 0.8) and did not significantly modify the coefficients of other included variables.

### Acute Rejection-Free Survival

Because there were few heterogenous events (*n* = 23), we did not analyze chronic rejection data and focused on acute rejection events. A total of 106 acute rejection events occurred during the follow-up, considering T-cell–medicated rejection, antibody-mediated rejection, and mixed acute rejection together: 38 (19%) and 70 (17%) events occurred in the AAV-GN and control groups, respectively (Table 2). In univariable analysis, there was no significant difference in the cumulative incidence of acute rejection when comparing the AAV-GN group to the control group (15% vs. 12% at 1 year, 18% vs. 17% at 5 years, 19% vs. 19% at 10 years, *P* = 0.8, Figure 2c). Results of the multivariable analysis are shown in Supplementary Table S7.

In the AAV-GN group, in multivariable analysis, the presence of ANCA at AAV-GN diagnosis (HR = 0.19 [0.04–0.87], *P* = 0.032) and KT maintenance treatment with AZA (HR = 3.73 [1.34–10.4], *P* = 0.012) were associated with acute rejection-free survival (Table 5). ANCA status was associated with acute rejection-free survival, but with discordant results according to the type of test used as follows: (i) when considering the maximum number of subjects with available testing (IIF or EIA, *n* = 154), ≥ 1 positive ANCA test tended to be associated (HR = 0.54 [0.25–1.15], *P* = 0.11); (ii) when considering IIF only (*n* = 130), positive status was associated (HR = 0.31 [0.12–0.81], *P* = 0.016); (iii) when considering EIA only (*n* = 112), no association were found; (iv) when considering patients with both IIF and EIA testing (*n* = 89), double positive ANCA (HR = 0.16 [0.04–0.74], *P* = 0.019) was associated (Supplementary Table S6, Supplementary Figure S4C). Again, the analysis was, however, limited because of a small number of events.

**Table 5:** Factors associated with acute rejection (in the AAV-GN cohort)

Characteristics	Univariable					Multivariable (simplified)				
	*n*	Event *n*	HR	95% CI	*P*-value	*n*	Event *n*	HR	95% CI	*P*-value
Baseline characteristics										
Male sex (vs. female)	203	38	0.91	0.46–1.79	0.8					
BMI (kg/m^2^)	203	38	1.00	0.92–1.08	>0.9					
Hypertension (vs. no)	201	37	0.42	0.20–0.87	0.020					
Diabetes (vs. no)	203	38	0.45	0.11–1.86	0.3					
Presentation at AAV-GN diagnosis										
Age (yrs)	198	38	1.03	1.00–1.06	0.062					
Kidney involvement (vs. no)	203	38	Inf.	Inf.	-					
Creatinine (μmol/l)	132	29	1.00	1.00–1.00	0.7					
Need for KRT within 30 d (vs. no)	159	30	1.00	0.48–2.08	>0.9					
Proteinuria (g/g)	119	22	1.00	0.86–1.16	>0.9					
Hematuria (vs. no)	126	25	0.30	0.09–1.01	0.051					
Lung involvement (vs. no)	184	36	0.64	0.30–1.36	0.2					
Heart involvement (vs. no)	183	36	1.70	0.41–7.09	0.5					
Neurological involvement (vs. no)	183	36	0.40	0.05–2.89	0.4					
ENT involvement (vs. no)	184	36	0.44	0.15–1.25	0.12					
Immunological findings										
Presence of ANCA (vs. no)	191	36	0.26	0.06–1.10	0.068	130	21	0.19	0.04–0.87	0.032
Anti-PR3 (vs. anti-MPO)	174	33	0.97	0.45–2.10	>0.9					
Therapeutic management of AAV-GN										
Induction remission therapy										
Plasma exchange (vs. no)	183	36	0.76	0.36–1.58	0.5					
Methylprednisolone pulses (vs. no)	182	34	0.87	0.26–2.86	0.8					
Prednisone (vs. no)	193	37	0.73	0.22–2.39	0.6					
Cyclophosphamide (vs. no)	190	36	0.92	0.42–2.03	0.8					
Rituximab (vs. no)	189	36	0.96	0.29–3.13	>0.9					
Maintenance therapy										
Prednisone (vs. no)	158	30	1.21	0.42–3.49	0.7					
Cyclophosphamide (vs. no)	175	32	0.79	0.24–2.61	0.7					
Rituximab (vs. no)	176	32	0.83	0.32–2.16	0.7					
Azathioprine (vs. no)	176	31	0.83	0.39–1.78	0.6					
Mycophenolic acid (vs. no)	176	32	1.15	0.47–2.80	0.8					
Outcomes before KT										
Relapse	196	37	0.84	0.39–1.78	0.6					
Preemptive transplantation	203	38	0.31	0.04–2.27	0.3					
Status at KT										
Recipient age (yrs)	203	38	1.03	0.99–1.06	0.13					
First transplantation	203	38	0.97	0.30–3.15	>0.9					
Calculated PRA	155	22	0.99	0.98–1.01	0.4					
ANCA status at KT										
According to IIF only - positive	140	22	0.28	0.11–0.72	0.008	130	21	0.31	0.12–0.81	0.016
According to EIA only - positive	118	25	0.71	0.32–1.57	0.4					
According to ≥ 1 test (IIF or EIA) - ≥ 1 positive	164	29	0.57	0.28–1.19	0.14					
According to both tests (IIF and EIA)										
Double negative			Ref.	-	-					
Discordant			0.43	0.14–1.33	0.14					
IIF positive & EIA negative			0.23	0.05–1.02	0.053					
IIF negative & EIA positive			2.86	0.62–13.2	0.2					
Double positive			0.14	0.03–0.64	0.011					
Transplantation procedure										
Donor age (yrs)	192	38	1.03	1.00–1.06	0.023					
Deceased donor (vs. no)	203	38	1.75	0.42–7.28	0.4					
Donor creatinine (per 50 μmol/l increment)	177	33	0.64	0.35–1.17	0.15					
Cold ischemia time (h)	202	38	1.00	0.96–1.05	>0.9					
HLA-mismatches (total)	202	38	1.23	1.00–1.52	0.054					
HLA-A	202	38	1.14	0.69–1.87	0.6					
HLA-B	202	38	1.36	0.77–2.40	0.3					
HLA-DR	202	38	1.45	0.88–2.40	0.15					
HLA-DQ	177	36	1.22	0.77–1.93	0.4					
Immunosuppressive regimen										
Induction therapy										
Anti-thymocyte globulin (vs. no)	203	38	0.60	0.28–1.28	0.2					
Basiliximab (vs. no)	203	38	1.75	0.83–3.72	0.14					
Prednisone (vs. no)	201	37	1.19	0.16–8.69	0.9					
Maintenance regimen										
Calcineurin inhibitors (vs. no)	203	38	Inf.	Inf.	-					
Mycophenolic acid (vs. no)	203	38	0.81	0.25–2.63	0.7					
Prednisone (vs. no)^a^	199	37	2.22	0.30–16.2	0.4					
mTOR inhibitors (vs. no)	198	38	1.56	0.79–3.06	0.2					
Azathioprine (vs. no)	200	38	3.01	1.32–6.88	0.009	130	21	3.73	1.34–10.4	0.012
Outcomes after KT										
DGF (vs. no)	201	38	1.52	0.69–3.33	0.3					

-, means not applicable; AAV-GN, ANCA-associated vasculitis with glomerulonephritis; ABMR, antibody-mediated rejection; ADPKD, autosomal dominant polycystic kidney disease; ANCA, antineutrophil cytoplasmic autoantibody; AR, acute rejection; BMI, body mass index; CI, confidence interval; DGF, delayed graft function; EIA, enzyme immunoassays; HR, hazard ratio; IIF, indirect immunofluorescence; KRT, kidney replacement therapy; KT, kidney transplantation; MPO, myeloperoxidase; mTOR, mammalian target of rapamycin; PR3, proteinase 3; PRA, panel reactive antibody; TCMR, T-cell–mediated rejection.

When there was not enough data in some subgroups, Cox regression model could not converge (result is given as "Inf.").

a Because KT maintenance regimen with steroids was differentially distributed between ANCA positive and negative patient, it was forced into an alternative multivariable model. Maintenance with steroid was not associated with acute rejection (HR = 0.90 [0.11–7.24], *P* > 0.9) and did not significantly modify the coefficients of other included variables.

### Overall Survival

A total of 131 deaths occurred during the follow-up as follows: 50 (24%) and 81 (20%) in AAV-GN and control group, respectively. In univariable analysis, overall survival was lower in the AAV-GN group (96% vs. 97% at 1 year, 83% vs. 86% at 5 years, 59% vs. 71% at 10 years, *P* = 0.03, Figure 2d). In multivariable analysis, AAV-GN were at higher risk of death (HR = 1.48 [1.03–2.13], *P* = 0.034, Supplementary Table S8).

In the AAV-GN group, in multivariable analysis, only recipient age at KT (HR = 1.06 [1.02–1.11]/ 1-yr increment, *P* = 0.004) and ANCA type at AAV-GN diagnosis (HR = 0.43 [0.19–0.96], *P* = 0.040) were associated with patient survival (Table 6). Of note, there was no difference in patient survival regarding ANCA status at KT (Table 6 and Supplementary Figure S4D).

### Variation of Outcomes Incidence According to Transplantation Period

As a sensitivity analysis, we assessed whether transplantation period (2005–2014 vs. 2015–2023) influenced outcomes incidence (Supplementary Figure S8). For allograft failure, cumulative incidence did not differ significantly between groups across periods, and no interaction was observed between disease group (AAV-GN vs. control) and transplant era (Gray’s test *P* = 0.2). Similarly, relapse incidence remained stable over time (Gray’s test *P* = 0.2), and although acute rejection incidence decreased in the more recent era, no significant group-by-period interaction was detected (*P* = 0.4).

In contrast, overall survival patterns changed over time, but not uniformly across groups. Among AAV-GN recipients, survival remained relatively stable between eras (5-year era: 86% vs. 81%). In the control group, however, survival declined in the more recent period (5-year era: 93% vs. 80%). Multivariable Cox regression confirmed a higher mortality risk in AAV-GN versus control during the 2005 to 2014 era (adjusted HR = 2.01 [1.27–3.18], *P* = 0.0048, adjusted for age and diabetes, as in Supplementary Table S8 for the overall comparison), whereas no difference was observed in the 2015 to 2023 cohort (adjusted HR = 1.01 [0.58–1.77], *P* = 0.98). These results were further supported by a significant interaction between disease group and transplant period (*P* = 0.039), reflecting a convergence in survival between groups, driven primarily by a decline in survival among controls.

### Delays Between Events and Association With Outcomes

Although the time between waitlisting and KT was lower for patients with AAV-GN (14 vs. 17 months, *P* = 0.044), the time between ESKD and KT (30 vs. 25 months, *P* = 0.023), and between ESKD and waitlisting (14 vs. 7 months, *P* < 0.001) was higher for patients with AAV-GN (Supplementary Figure S3 and Supplementary Table S9).

We then studied the impact of delays on the previously described events. When focusing on the AAV-GN group, in univariable analysis, there was no clear trend of an impact of the various considered delays on graft survival, relapse-free survival, acute rejection-free survival, and patient survival (Supplementary Figure S9). Some analyses were, however, lacking power because of the low number of patients with short delays (< 6 months or < 12 months) between these time points.

## Discussion

In this study, we analyzed KT outcomes in a large, predominantly Caucasian cohort of patients with AAV-GN, compared with a matched nonvasculitis ESKD control group. Short-term outcomes (including DGF, kidney function recovery, and acute rejection) were similar between groups. In contrast, long-term outcomes were poorer in patients with AAV-GN, with higher rates of graft failure and overall mortality. ANCA positivity at the time of transplantation was associated with immune events, including both relapse and reduced rejection risk. In addition, as observed in other kidney diseases, the use of AZA as maintenance therapy was linked to an increased risk of rejection and inferior graft survival.

First, we showed that there was no difference in DGF frequency and estimated glomerular filtration rate evolution over time (up to 5 years post-KT). However, in multivariable analysis, there was a trend of a poorer long-term graft survival in patients with AAV-GN when compared with the control group. This is in line with a previous study from the Australia and New Zealand Dialysis and Transplant registry that found a lower kidney survival in patients with microscopic polyangiitis (but not for patients with granulomatosis with polyangiitis) when compared with the control group^20^; however these results were in disagreement with the data from European Renal Association^30^ and UNOS registry,^15^^,^^21^ which found a better kidney survival in patients with AAV. However, patients from the European Renal Association registry were younger (median age: 55 years, vs. 59 years in our cohort) and much more frequently transplanted from a living donor (29% vs. 10% in our cohort). Patients from the UNOS registry were also much younger (mean age at KT < 50 years) with 45% of kidneys from living donors. Of note, some smaller studies did not find a difference in allograft survival between AAV-GN and a control group.^13^^,^^14^ The definition and characteristics of the control group may explain these variations between studies. Several hypotheses, albeit speculative, may be proposed to explain the observed trend toward poorer graft survival in patients with AAV-GN. These include persistent subclinical microvascular inflammation, a higher burden of cardiovascular comorbidities (potentially exacerbated by prolonged dialysis exposure and the cumulative effects of previous immunosuppressive therapies), and the possible use of immunosuppression minimization strategies in more frail recipients.

In our cohort, the 5-year cumulative incidence of graft failure reached 12% for AAV-GN, congruent with recent studies performed in Europe countries,^12^^,^^16^ and with data from the French REIN registry.^7^ In this study that included all French patients with AAV-GN with ESKD on the waiting list since 2002, we showed the benefit of transplantation for patients with AAV-GN (in comparison to waiting in chronic dialysis) and found a 5-year graft survival of 87%.

We identified maintenance with AZA treatment as a major determinant of graft failure, both in the entire cohort and in the AAV-GN cohort. Following current international guidelines, this reinforces the fact that treatment with AZA should be avoided posttransplantation when possible, including in the AAV-GN population, even if it was shown to be superior to mycophenolate mofetil, outside the transplant context, for maintaining AAV remission.^31^ As expected, immunological events occurring on the transplant (relapses or rejection) were strongly associated with graft survival.

Second, we observed that posttransplant relapses were infrequent, occurring in < 8% of patients with AAV-GN. Despite this low incidence, our study is one of the largest to date reporting such a number of relapse events after KT in this population.^32^^,^^33^ As expected, relapses were strongly associated with subsequent allograft loss. These episodes involved both renal and extrarenal manifestations, consistent with previous reports.^12^^,^^34^ In our cohort, 2 factors emerged as potential determinants of relapse: absence of ANCA at initial AAV-GN diagnosis and ANCA positivity at the time of transplantation. The association with ANCA negativity at diagnosis should be interpreted with caution, given the small number of ANCA-negative patients in our cohort. In our analysis, a nearly significant association was observed when ANCA positivity was defined by either IIF or EIA positivity, with the signal primarily driven by IIF. However, interpretation is limited by the low number of relapse events and potential variability in IIF methodology between centers, including differences in thresholds for positivity. This data align with previously published studies, although the literature remains divided on this issue, with some studies reporting a significant association^11^^,^^14^^,^^27^^,^^35^ and others not confirming such a link.^36^^,^^37^ Of note, we found no interaction between ANCA status at transplantation and ANCA subtype (myeloperoxidase vs. proteinase 3) with respect to relapse risk.

Third, in the modern era of immunosuppressive therapy (i.e., most patients were treated with a calcineurin inhibitor and mycophenolate–based immunosuppression regimen),^38^ we found a similar rate of acute rejection between groups. In patients with AAV-GN, ANCA positivity at AAV-GN diagnosis was associated with a decreased risk of acute rejection. In this setting, ANCA positivity may have influenced clinicians to keep immunosuppression stronger and/or longer. This reinforces the idea that ANCA negative patients have a vasculitis that is not the same as those of ANCA positive patients.^39^^,^^40^ Relapse risk (before KT) of seronegative patients with AAV has been reported inconsistently.^40^, ^41^, ^42^ Lastly, maintenance therapy post-KT with AZA was again associated with worse outcomes, increasing the risk of acute rejection without decreasing the risk of relapses. This is congruent with the general recommendation on post-KT immunosuppressive management.^43^, ^44^, ^45^

Fourth, we found that overall survival in patients with AAV-GN was poorer than in controls, with an adjusted increased risk of death approaching +50% over the entire study period. Unfortunately, we did not have access to cause-of-death data to identify the drivers of this excess mortality (whether vasculitis-related, increased infection-related, cardiovascular complications, or malignancies). As with graft survival, this finding is in line with data from the Australia and New Zealand Dialysis and Transplant registry,^20^ which reported lower survival in patients with microscopic polyangiitis but not patients with granulomatosis with polyangiitis, whereas other studies based on younger and predominantly granulomatosis with polyangiitis populations (e.g., from the European Renal Association^30^ and UNOS registries^15^^,^^21^) found more favorable outcomes.

Importantly, when stratifying our cohort by transplant era, we observed that this survival disparity between AAV-GN and control recipients was limited to the earlier era (2005–2014). In the more recent transplant period (2015–2023), overall survival in patients with AAV-GN remained stable and was no longer significantly different from controls. This suggests that the survival gap between AAV-GN and nonvasculitis recipients has narrowed over time, possibly reflecting improvements in vasculitis management, transplant care, and widened patient selection. Still, older patients with AAV-GN may remain more vulnerable to long-term complications (whether due to cumulative immunosuppression, chronic inflammation, or delayed access to transplantation) compared with similarly aged controls without autoimmune disease. In our cohort, the overall 5-year survival rate for patients with AAV-GN reached 83%, which is consistent with recent observational studies.^11^^,^^12^^,^^16^^,^^33^

In our cohort, very few patients received a KT within 12 months, or even within 24 months from AAV-GN diagnosis. In addition, data regarding the exact date of AAV remission was not available for most of them. We then could not verify the increased risk of death in patients transplanted within 12 months from remission that was found by Little *et al.*^16^ and which led the Kidney Disease: Improving Global Outcomes to recommend a minimum of 6 months between remission and transplantation.^46^ Such short delays are, however, rarely a problem in real life; when considering specifically waitlisted patients with AAV-GN, we have previously shown that cumulative incidence of transplantation reaches 4%, 10% ,and 27% by 1, 2, and 5 years, respectively from ESKD onset.^7^

Our study has several limitations, mostly because of its retrospective design that may have contributed to missing data, especially regarding ANCA status at transplantation. In addition, the absence of data on the exact date of AAV-GN remission prevented us from analyzing the interval between remission and transplantation (a known knowledge gap in the field). This reflects a common challenge in retrospective vasculitis studies, given the lack of standardized remission definitions and the inconsistent documentation across centers and time periods. Standardized definitions and prospective collection of remission dates will be essential to definitively address this important question. However, if the ASTRE database was used to identify patients with AAV-GN transplanted, all data were manually and carefully gathered, on each patient's record on a detailed scale. In additon, a small rate of AAV relapse after KT precluded stronger statistical analysis, especially when looking at the role of ANCA status at transplantation. Finally, a centralized histological review of biopsy specimens applying the most recent Banff classification (including molecular diagnostics) could help identify specific features of rejection lesions in patients with AAV-GN, potentially allowing for more tailored therapeutic strategies in the future. Nonetheless, this is, to the best of our knowledge, the largest French experience with KT for AAV-GN, and one of the largest studies on this topic so far.^32^^,^^33^

In conclusion, this study shed light on the management and outcome of patients after KT in the context of AAV-GN. First, ANCA positivity at the time of KT is associated with immunological events (relapses and rejection) but not with patient and graft survival. Systematically evaluating ANCA status before KT is thus recommended to help predict upcoming events but shall not preclude the transplant procedure. Second, we did not identify an ideal timing after AAV-GN diagnosis or ESKD onset to prevent upcoming events (we were limited by few early transplantation procedures) but noticed that patients with AAV-GN were waitlisted and transplanted later than the control patients, highlighting the need for improved access to transplantation. Future prospective studies with standardized definition of remission are needed to better address the question of optimal timing for KT. Lastly, the use of AZA as maintenance therapy for KT was independently associated with graft survival and rejection (without protecting from relapses), thus encouraging the reconsideration of posttransplant use of this molecule (to be used in very specific cases, e.g., intolerance to mycophenolate mofetil), thus following current international guidelines for other nephropathies. Overall, KT is a valuable option for patients with AAV-GN: the relapse rate is low, and the occurrence of acute rejection is comparable to a population of control patients. However, graft and patient survival were lower in AAV-GN when compared with control patients, advocating for a specific posttransplant follow-up. The burden of pretransplant treatment and events, and their impact on posttransplant outcomes must definitely be considered when managing these patients.

## Disclosure

All the authors declared no competing interests.

## Supplementary Material

Supplementary File (PDF) **Figure S1.** Matching between AAV-GN cases and control patients. **Figure S2.** ANCA status at the time of KT, according to EIA and/or IIF. **Figure S3.** Distribution of delays (time between diagnosis, ESKD, waitlisting and kidney transplantation). **Figure S4.** Event-free survival after kidney transplantation according to ANCA status at the time of transplantation (in the AAV-GN subgroup). **Figure S5.** Kidney involvement and management of relapsing patients (in the AAV-GN subgroup). **Figure S6.** Event-free survival according to relapsing status (in the AAV-GN subgroup). **Figure S7.** Relapse-free survival according to ANCA subtype at diagnosis and ANCA status at the time of KT (in the AAV-GN subgroup). **Figure S8.** Event-free survival after kidney transplantation according to transplantation period. **Figure S9.** Impact of delays on event-free survival after kidney transplantation (in the AAV-GN subgroup). **Table S1.** Comparison of patients with AAV-GN according to ANCA status at KT. **Table S2.** Factors associated with DGF (in the AAV-GN cohort). **Table S3.** Factors associated with graft failure (in the whole cohort). **Table S4.** Description of patients with AAV-GN according to relapsing status. **Table S5.** Description of each AAV-GN relapse. **Table S6.** Associated between ANCA status at KT, relapses and acute rejection (multivariable models) (in the AAV-GN cohort). **Table S7.** Factors associated with acute rejection (in the whole cohort). **Table S8.** Factors associated with death (in the whole cohort). **Table S9.** Distribution of delays between diagnosis, ESKD, waitlisting and kidney transplantation.

## Footnotes

Supplementary File (PDF)
**Figure S1.** Matching between AAV-GN cases and control patients. **Figure S2.** ANCA status at the time of KT, according to EIA and/or IIF. **Figure S3.** Distribution of delays (time between diagnosis, ESKD, waitlisting and kidney transplantation). **Figure S4.** Event-free survival after kidney transplantation according to ANCA status at the time of transplantation (in the AAV-GN subgroup). **Figure S5.** Kidney involvement and management of relapsing patients (in the AAV-GN subgroup). **Figure S6.** Event-free survival according to relapsing status (in the AAV-GN subgroup). **Figure S7.** Relapse-free survival according to ANCA subtype at diagnosis and ANCA status at the time of KT (in the AAV-GN subgroup). **Figure S8.** Event-free survival after kidney transplantation according to transplantation period. **Figure S9.** Impact of delays on event-free survival after kidney transplantation (in the AAV-GN subgroup). **Table S1.** Comparison of patients with AAV-GN according to ANCA status at KT. **Table S2.** Factors associated with DGF (in the AAV-GN cohort). **Table S3.** Factors associated with graft failure (in the whole cohort). **Table S4.** Description of patients with AAV-GN according to relapsing status. **Table S5.** Description of each AAV-GN relapse. **Table S6.** Associated between ANCA status at KT, relapses and acute rejection (multivariable models) (in the AAV-GN cohort). **Table S7.** Factors associated with acute rejection (in the whole cohort). **Table S8.** Factors associated with death (in the whole cohort). **Table S9.** Distribution of delays between diagnosis, ESKD, waitlisting and kidney transplantation.
