# Supplementary material for: Outcomes After Kidney Transplantation in Antineutrophil Cytoplasmic Autoantibody–Associated Renal Vasculitis
Source: Kidney Int Rep. 2025 Jul 14;10(10):3465–83. doi: 10.1016/j.ekir.2025.07.008 (PMC12546434; doi:10.1016/j.ekir.2025.07.008)
Supplement: Supplementary File (PDF) — Figure S1. Matching between AAV-GN cases and control patients. Figure S2. ANCA status at the time of KT, according to EIA and/or IIF. Figure S3. Distribution of delays (time between diagnosis, ESKD, waitlisting and kidney transplantation). Figure S4. Event-free survival after kidney transplantation according to ANCA status at the time of transplantation (in the AAV-GN subgroup). Figure S5. Kidney involvement and management of relapsing patients (in the AAV-GN subgroup). Figure S6. Event-free survival according to relapsing status (in the AAV-GN subgroup). Figure S7. Relapse-free survival according to ANCA subtype at diagnosis and ANCA status at the time of KT (in the AAV-GN subgroup). Figure S8. Event-free survival after kidney transplantation according to transplantation period. Figure S9. Impact of delays on event-free survival after kidney transplantation (in the AAV-GN subgroup). Table S1. Comparison of patients with AAV-GN according to ANCA status at KT. Table S2. Factors associated with DGF (in the AAV-GN cohort). Table S3. Factors associated with graft failure (in the whole cohort). Table S4. Description of patients with AAV-GN according to relapsing status. Table S5. Description of each AAV-GN relapse. Table S6. Associated between ANCA status at KT, relapses and acute rejection (multivariable models) (in the AAV-GN cohort). Table S7. Factors associated with acute rejection (in the whole cohort). Table S8. Factors associated with death (in the whole cohort). Table S9. Distribution of delays between diagnosis, ESKD, waitlisting and kidney transplantation. [file mmc1.pdf]

Outcomes after kidney transplantation in ANCA-associated renal vasculitis

M. Dekervel[1*], P. Traversat[1*], P. Gatault[2], L. Golbin[3], A. Thierry[4], V. Chatelet[5], S. Caillard[6], A. Duval[6], E. Cornec-Le Gall[7], M. Planchais[1], A. Duveau[1], D. Bertrand[8], JP. Rerolle[9], C. Garrouste[10], D. Anglicheau[11], M. Jaureguy[12], F. Duthe[4], JF. Augusto[1,13], B. Brilland[1,13].

Supplementary Material.

Supplementary Figures (PDF)

Supplementary Tables (PDF)

Supplementary Figure and Table legends.

Supplementary Figure S1 – Matching between AAV-GN cases and control patients.

Matching quality regarding center (A), year of transplantation (B), sex (C) and recipient age (D). AAV-GN cases were considered well-matched with controls if they came from the same center (A), were transplanted during the same period (± 5 years, B), were of the same sex (C) and within the same age range (± 5 years, D).

Abbreviations: AAV-GN, ANCA-associated vasculitis with glomerulonephritis.

Supplementary Figure S2 – ANCA status at the time of KT, according to EIA and/or IIF.

Abbreviations: EIA, enzyme immunoassays; IIF, indirect immunofluorescence; NA: not available.

Supplementary Figure S3 – Distribution of delays (time between diagnosis, ESKD, waitlisting and kidney transplantation).

A. Schematic of delays between AAV-GN diagnosis (when applicable), ESKD, waitlisting, and kidney transplantation in the AAV-GN (red) and control (green) groups.

B. Delays between AAV-GN diagnosis (when applicable), ESKD, waitlisting, and kidney transplantation in the AAV-GN (red) and control (green) groups ; as continuous variable.

C. Delays between AAV-GN diagnosis (when applicable), ESKD, waitlisting, and kidney transplantation in the AAV-GN (red) and control (green) groups ; as 3 categories variable.

Abbreviations: AAV-GN, ANCA-associated vasculitis with glomerulonephritis ; CTRL, control ; Dg, diagnosis ; ESKD, end-stage kidney disease ; KT, kidney transplantation ; WL, waitlisting.

Supplementary Figure S4 – Event-free survival after kidney transplantation according to ANCA status at the time of transplantation (in the AAV-GN subgroup).

Survival free of ESKD (A), relapse (B), acute rejection (C) or death (D).

Upper panel, ANCA positivity defined by positivity in IIF or EIA (n = 167).

Lower panel, ANCA positivity defined by ANCA status (double negative, discordant/simple positive, double positive) in both IIF and EIA (n = 97). See **Supplementary Figure S2** for distribution of ANCA status according to the test that was used.

Abbreviations: AAV-GN, ANCA-associated vasculitis with glomerulonephritis; EIA, enzyme immunoassays; IIF, indirect immunofluorescence

Supplementary Figure S5 – Kidney involvement and management of relapsing patients (in the AAV-GN subgroup).

Kidney involvement (A) and management (B) of relapsing patients.

Relapses often involved the kidney allograft (12/15, 80%) and most had acute kidney injury (10/12, 83%), proteinuria (8/11, 73%) or hematuria (8/12, 67%). All relapsing patients with kidney involvement (12/15, 80%) underwent confirmatory biopsy. Berden's histopathological classification was available in 10 of them, showing mixed lesions (class III) for most of them (4/10, 40%). Relapses involved other organs in 7/15 (47%) cases: 4, 3 and 2 patients had lung, ENT and joint involvement, respectively. Treatment consisted of change/addition of immunosuppressive therapy (13/15, 87%) and increase/addition of steroids (12/15, 80%) for most of them. A small proportion were treated with plasma exchange (5/15, 33%) or intravenous immunoglobulin (2/15, 13%).

Supplementary Figure S6 – Event-free survival according to relapsing status (in the AAV-GN subgroup).

Survival free of ESKD (A), or death (B) according to any relapse status.

Survival free of ESKD (C), or death (D) according to renal relapse status.

For panel A: because one patient experienced a relapse (not involving the transplant) after graft failure (see Supplementary Table S4), he was considered as a non-relapser for this specific analysis only.

Supplementary Figure S7 – Relapse-free survival according to ANCA subtype at diagnosis and ANCA status at the time of KT (in the AAV-GN subgroup).

A) ANCA positivity defined by positivity in IIF or EIA (total number of AAV patients = 145).

B) ANCA positivity defined by ANCA positivity defined by ANCA status (double negative, discordant/simple positive, double positive) in both IIF and EIA (total number of AAV patients = 83).

Supplementary Figure S8 – Event-free survival after kidney transplantation according to transplantation period.

Cumulative incidence of graft failure (A), relapses (B) or acute rejection (C), and overall survival (D), according to transplantation period (2005-2014 vs. 2015-2023).

Abbreviations: AAV-GN, ANCA-associated vasculitis with glomerulonephritis.

Supplementary Figure S9 – Impact of delays on event-free survival after kidney transplantation (in the AAV-GN subgroup).

For dichotomous variables (> 6 months, > 12 months, > 24 months), the shorter delay was considered as reference. For 3 or 5 categorical variables, the center modality was considered as reference.

Abbreviations: Dg, diagnosis ; ESKD, end-stage kidney disease ; KT, kidney transplantation ; WL, waitlisting.

Supplementary Figure S1

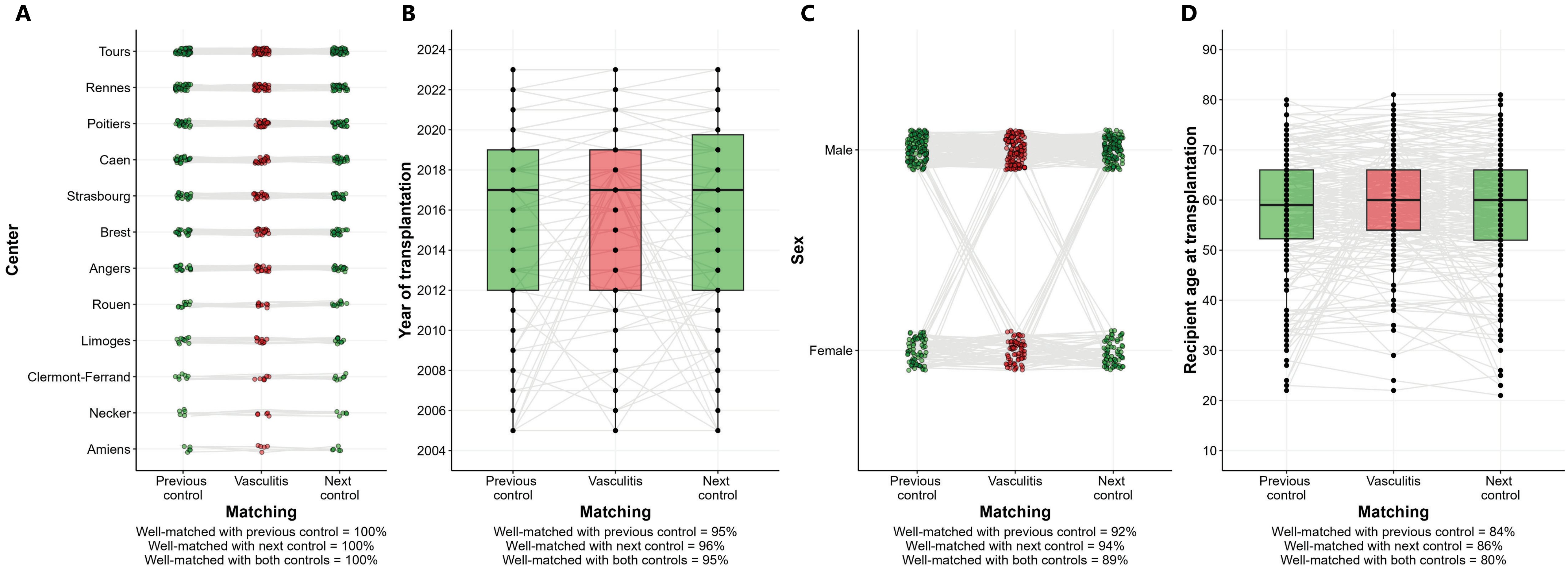

Supplementary Figure S2

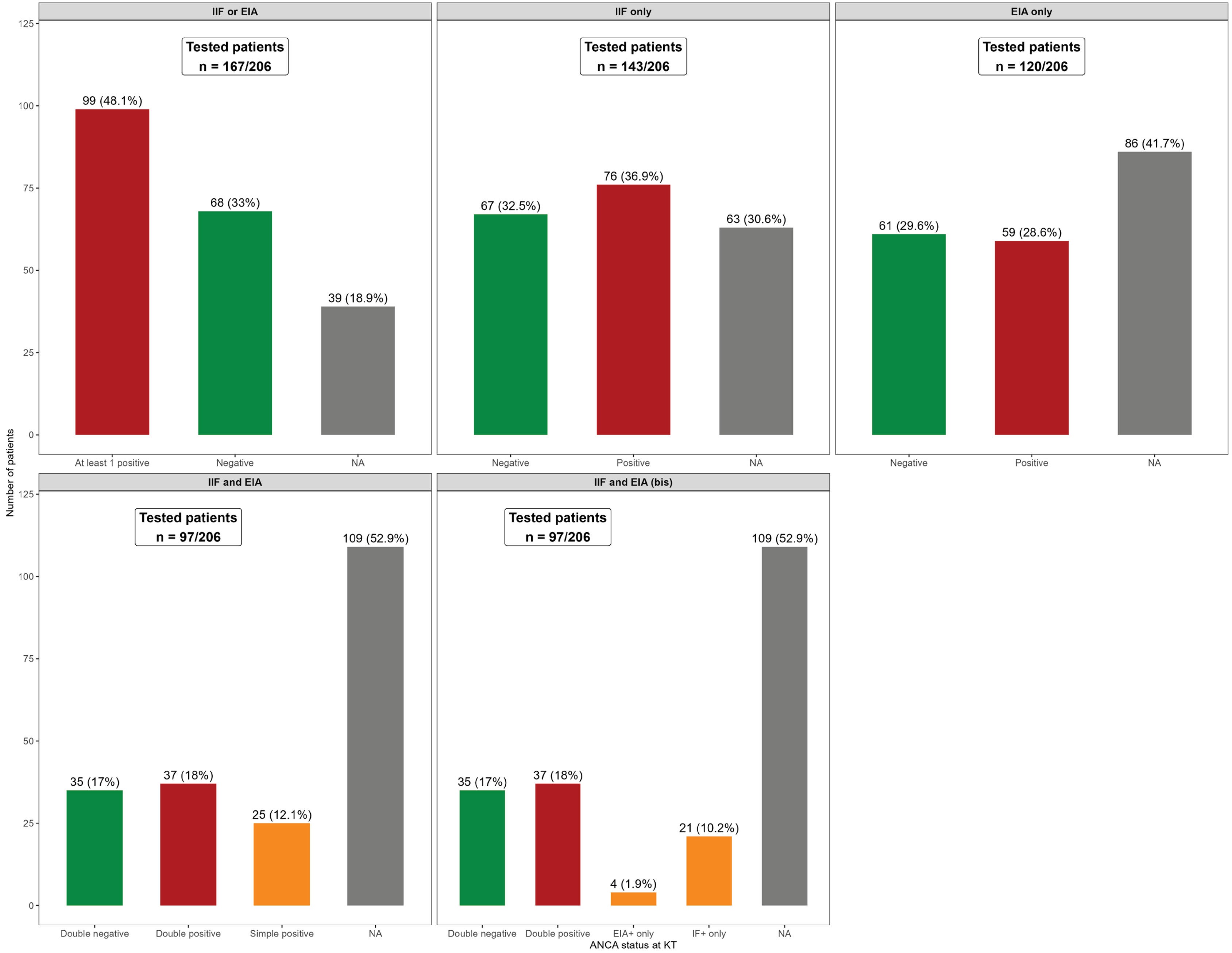

Supplementary Figure S3

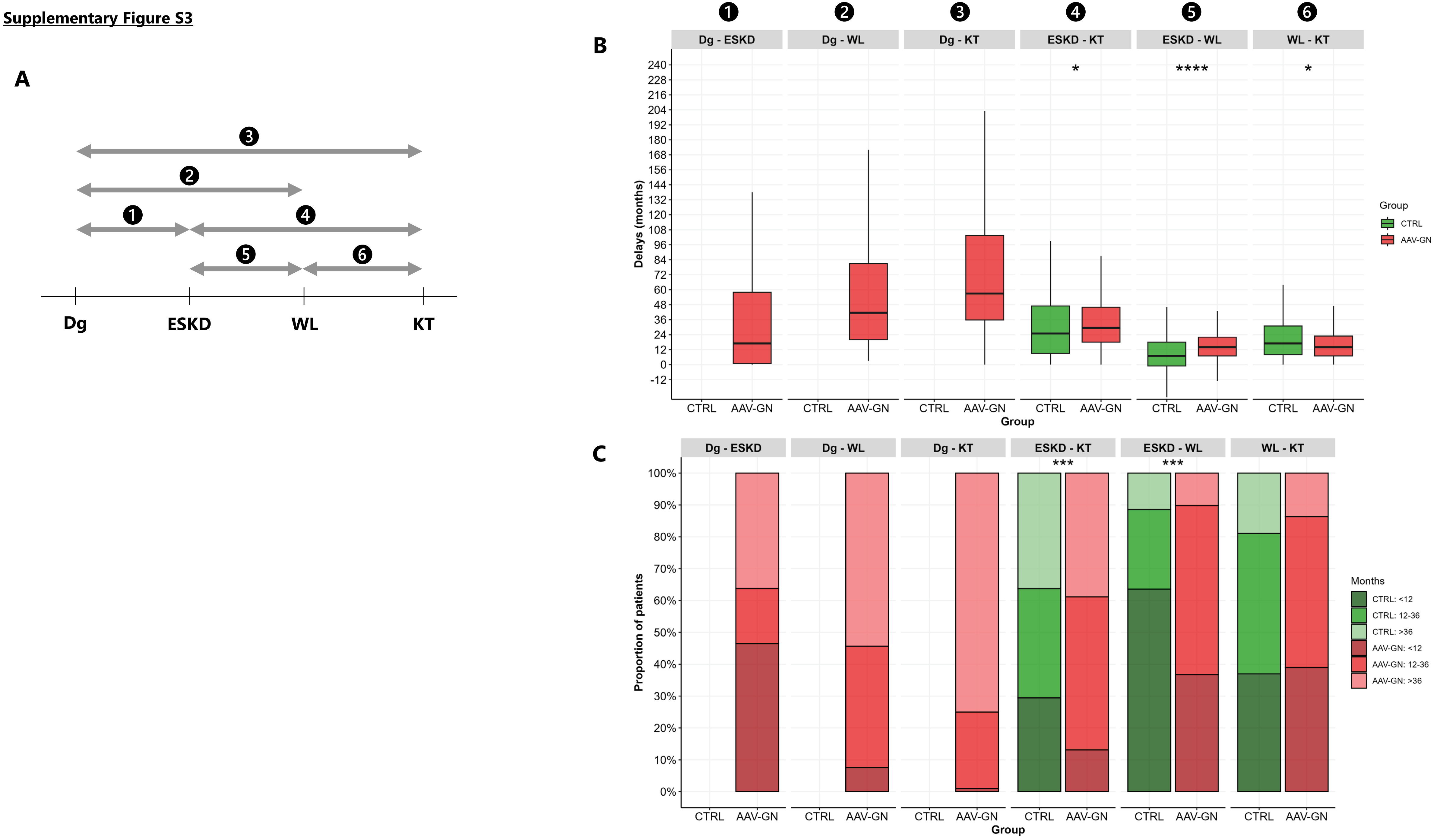

Supplementary Figure S4

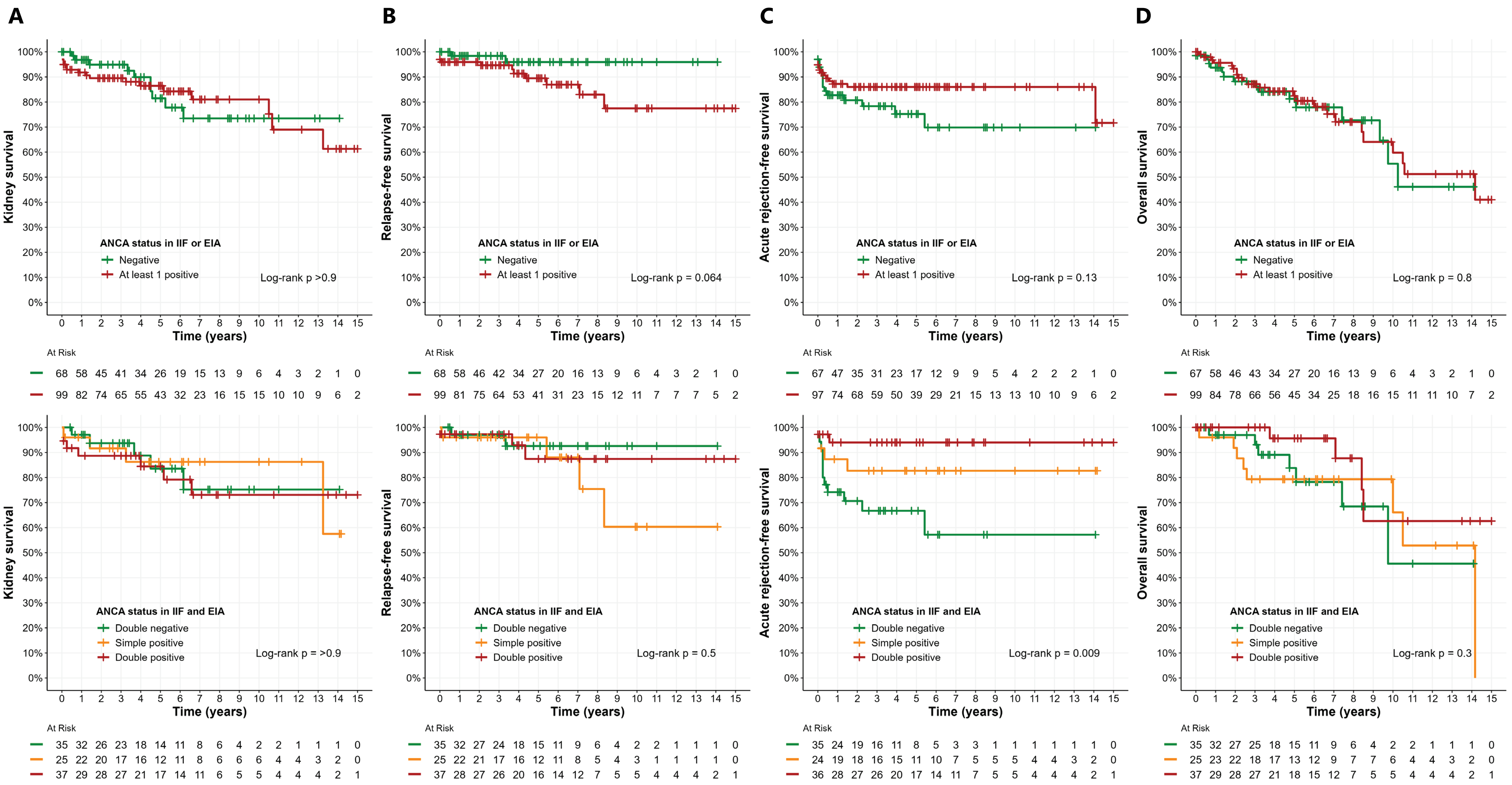

Supplementary Figure S5

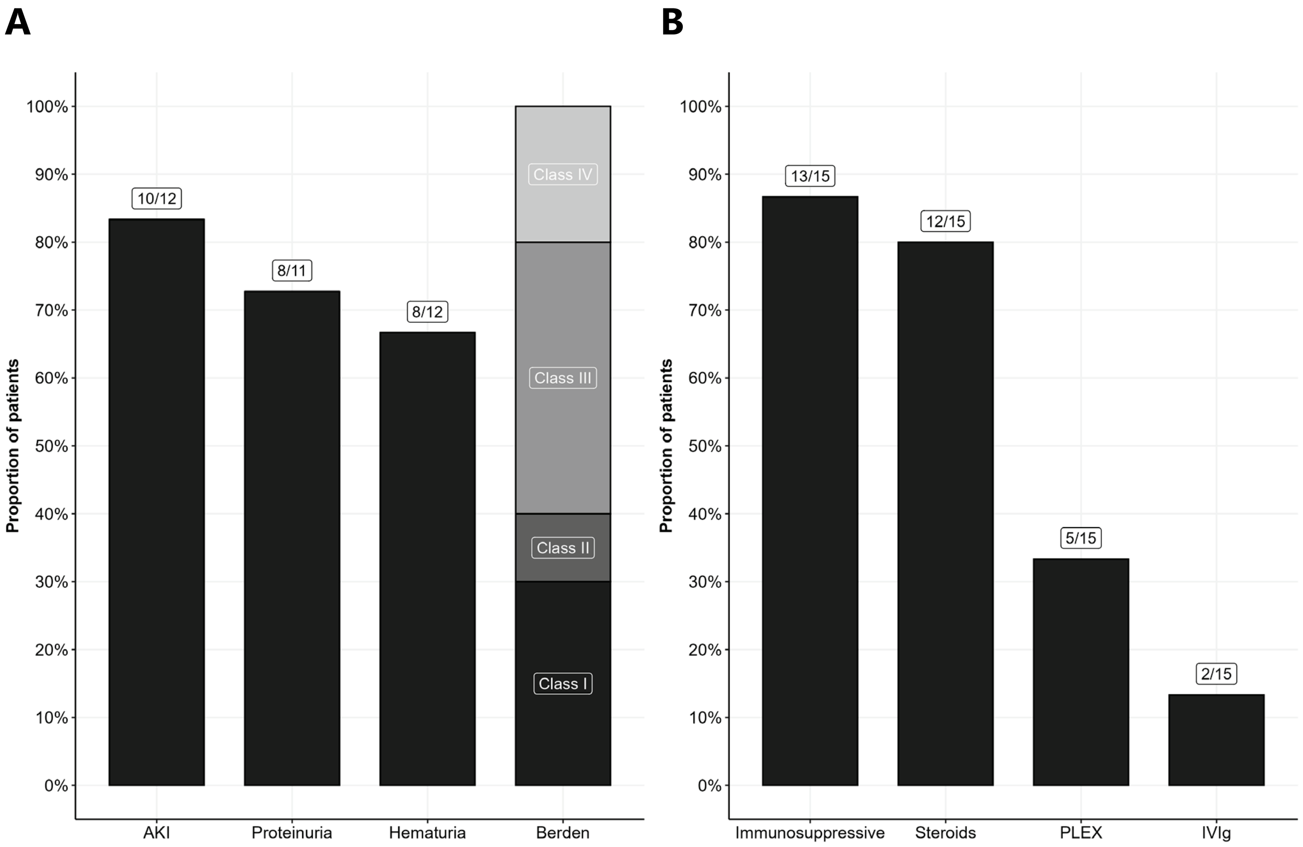

Supplementary Figure S6

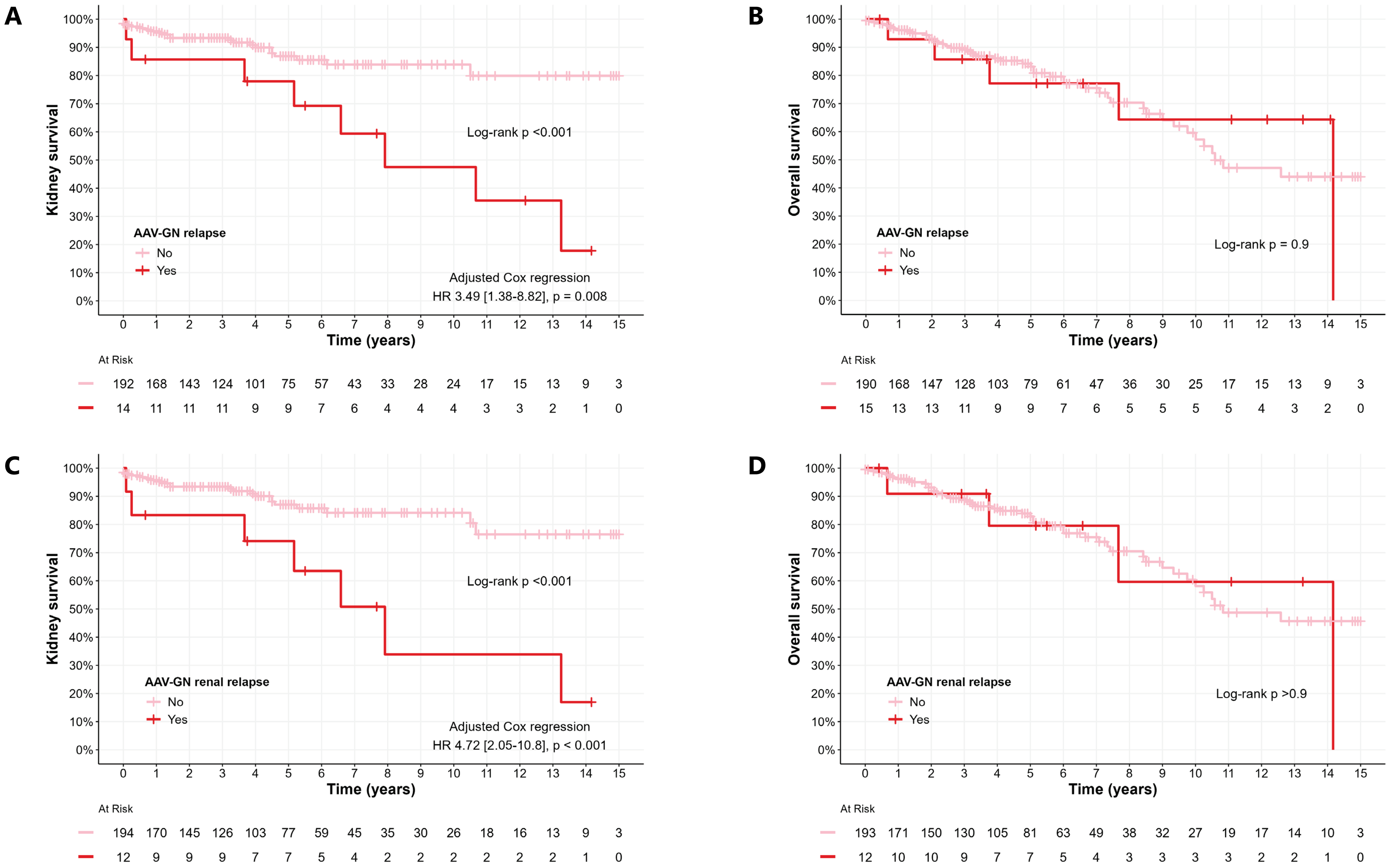

Supplementary Figure S7

A

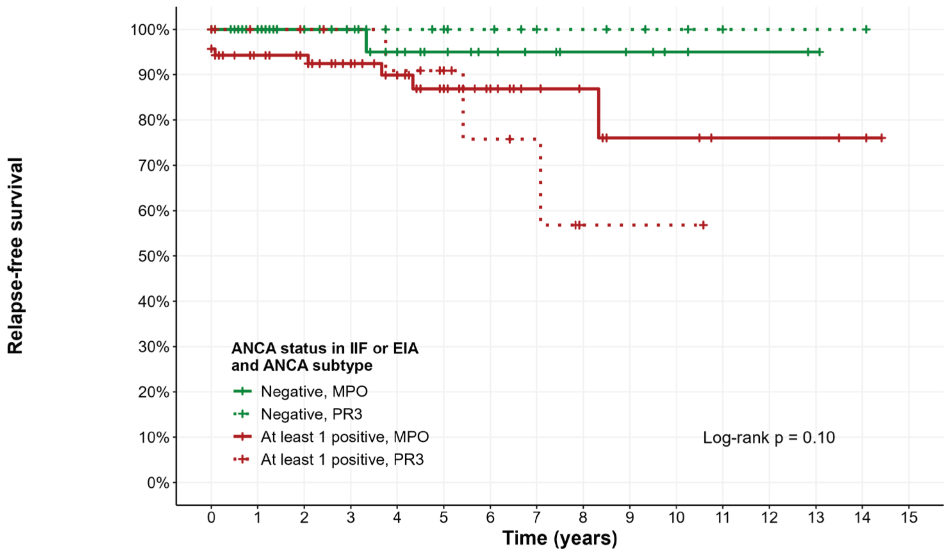

At Risk

	0	1	2	3	4	5	6	7	8	9	10	11	12	13	14	15
Negative, MPO	37	29	23	22	17	13	10	8	6	5	3	2	2	1	0	0
Negative, PR3	22	21	16	13	11	10	8	6	5	4	3	2	1	1	1	0
At least 1 positive, MPO	70	57	52	42	34	24	17	11	8	5	5	3	3	3	2	0
At least 1 positive, PR3	16	13	12	11	10	8	5	4	1	1	1	0	0	0	0	0

B

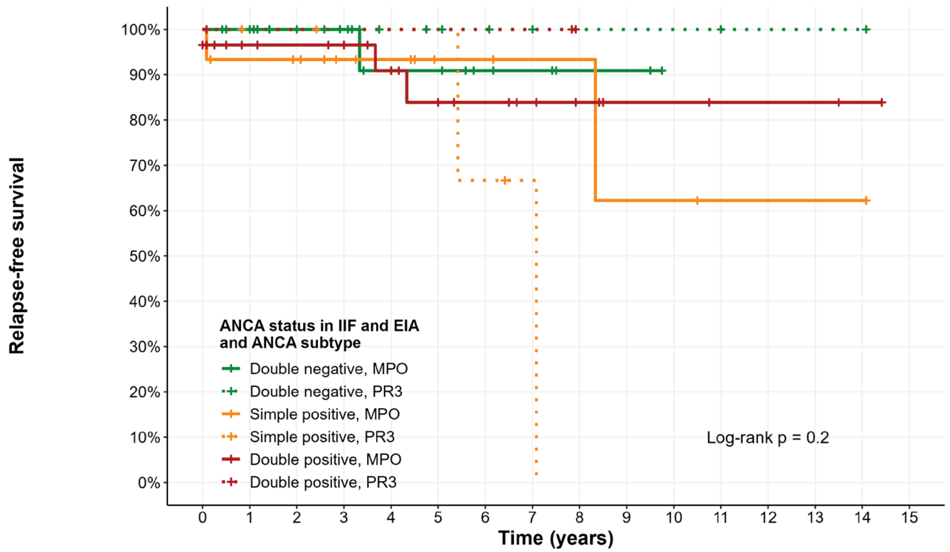

At Risk

	0	1	2	3	4	5	6	7	8	9	10	11	12	13	14	15
Double negative, MPO	18	16	14	13	9	8	5	4	2	2	0	0	0	0	0	0
Double negative, PR3	13	13	10	8	6	5	4	3	2	2	2	2	1	1	1	0
Simple positive, MPO	15	13	12	9	8	4	4	3	3	2	2	1	1	1	1	0
Simple positive, PR3	5	4	4	3	3	3	2	1	0	0	0	0	0	0	0	0
Double positive, MPO	29	22	21	20	16	12	10	8	5	3	3	2	2	2	1	0
Double positive, PR3	3	2	2	2	2	2	2	2	0	0	0	0	0	0	0	0

Supplementary Figure S8

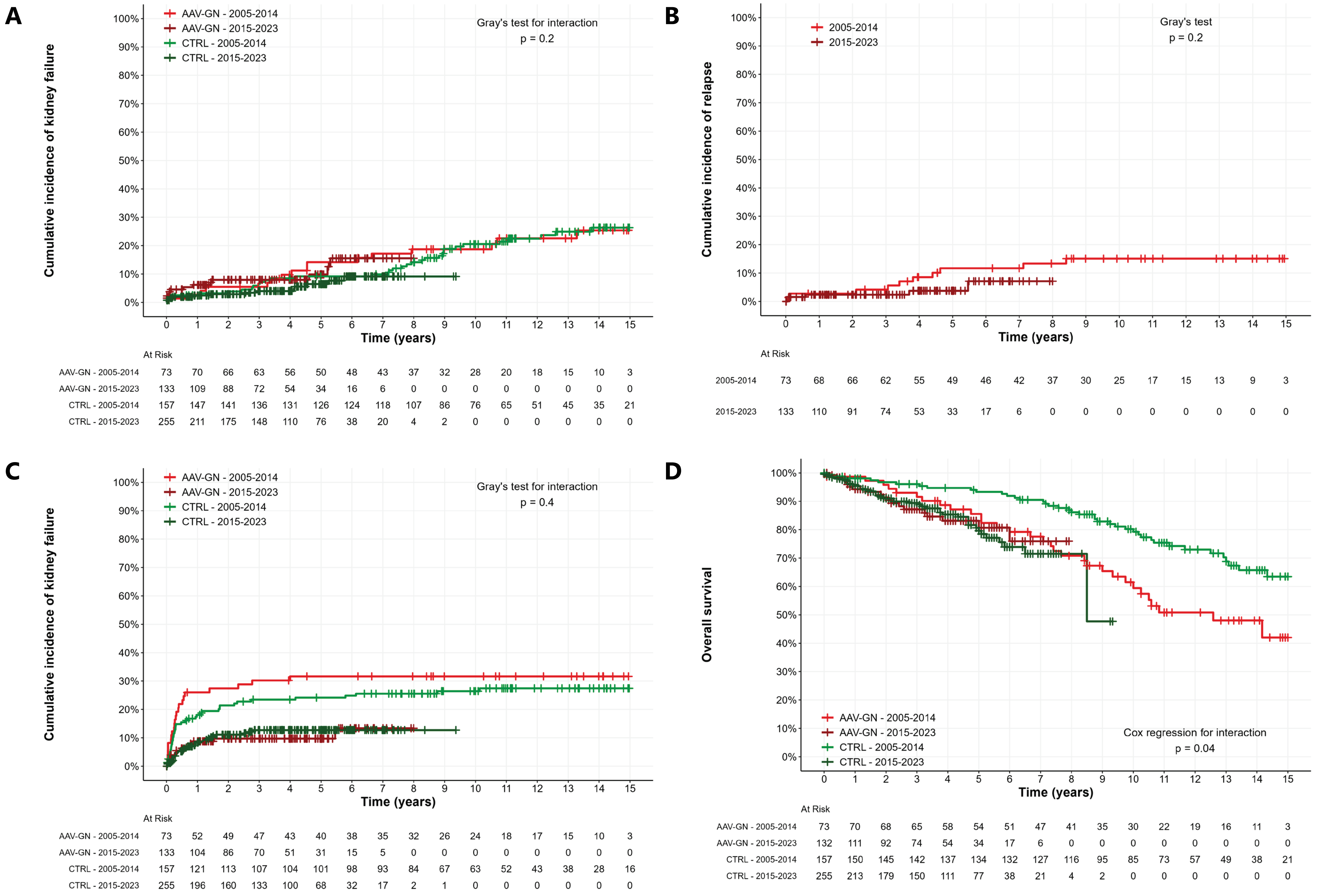

Supplementary Figure S9

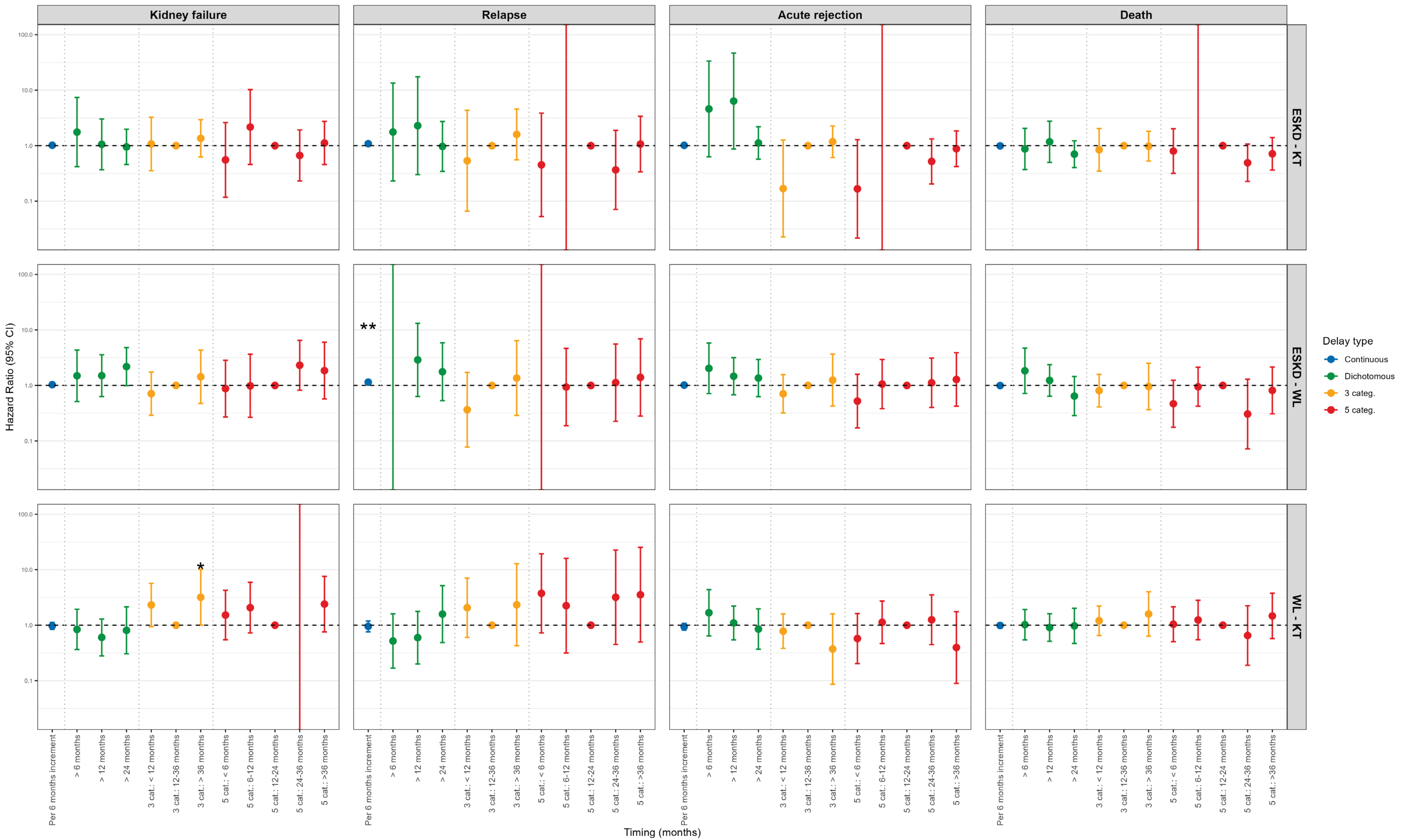

Supplementary Table S1 – Comparison of AAV-GN patients according to ANCA status at KT

Abbreviations: ABMR, antibody mediated rejection; ADPKD, autosomal dominant polycystic kidney disease; AR, acute rejection; BMI, body mass index; DGF, delayed graft function; EIA, enzyme immunoassays; IIF, indirect immunofluorescence; KRT, kidney replacement therapy; KT, kidney transplantation; PRA, panel reactive antibody; TCMR, T-cell mediated rejection.

"-" means not applicable. When there was not enough data in some subgroup, Cox regression model could not converge (result is given as "Inf.")

Supplementary Table S2 – Factors associated with DGF (in the AAV-GN cohort)

Abbreviations: ABMR, antibody mediated rejection; AR, acute rejection; BMI, body mass index; DGF, delayed graft function; EIA, enzyme immunoassays; IIF, indirect immunofluorescence; KRT, kidney replacement therapy; KT, kidney transplantation; PRA, panel reactive antibody; TCMR, T-cell mediated rejection.

"-" means not applicable. When there was not enough data in some subgroups, Cox regression model could not converge (result is given as "Inf.")

Supplementary Table S3 – Factors associated with graft failure (in the whole cohort)

Abbreviations: ABMR, antibody mediated rejection; AR, acute rejection; BMI, body mass index; DGF, delayed graft function; EIA, enzyme immunoassays; IIF, indirect immunofluorescence; KRT, kidney replacement therapy; KT, kidney transplantation; PRA, panel reactive antibody; TCMR, T-cell mediated rejection.

"-" means not applicable. When there was not enough data in some subgroups, Cox regression model could not converge (result is given as "Inf.")

Supplementary Table S4 – Description of AAV-GN patients according to relapsing status

Abbreviations: ABMR, antibody mediated rejection; AR, acute rejection; BMI, body mass index; DGF, delayed graft function; EIA, enzyme immunoassays; IIF, indirect immunofluorescence; KRT, kidney replacement therapy; KT, kidney transplantation; PRA, panel reactive antibody; TCMR, T-cell mediated rejection.

"-" means not applicable.

Supplementary Table S5 – Description of each AAV-GN relapse

Abbreviations: ATG, anti-thymocyte globulin; AZA, azathioprine; BSX, basiliximab; CNI, calcineurin inhibitors; CYC, cyclophosphamide; F, female; KT, kidney transplantation; M, male; MMF, mycophenolate mofetil; PLEX, plasma exchange; RTX, rituximab; Ster, steroids. "-" means not available.

Supplementary Table S6 – Associated between ANCA status at KT, relapses and acute rejection (multivariable models) (in the AAV-GN cohort)

Abbreviations: EIA, enzyme immunoassays; IIF, indirect immunofluorescence.
"-" means not applicable. When there was not enough data in some subgroups, Cox regression model could not converge (result is given as "Inf.")

Supplementary Table S7 – Factors associated with acute rejection (in the whole cohort)

Abbreviations: ABMR, antibody mediated rejection; ADPKD, autosomal dominant polycystic kidney disease; AR, acute rejection; BMI, body mass index; DGF, delayed graft function; EIA, enzyme immunoassays; IIF, indirect immunofluorescence; KRT, kidney replacement therapy; KT, kidney transplantation; PRA, panel reactive antibody; TCMR, T-cell mediated rejection.
"-" means not applicable. When there was not enough data in some subgroup, Cox regression model could not converge (result is given as "Inf.")

Supplementary Table S8 – Factors associated with death (in the whole cohort)

Abbreviations: ABMR, antibody mediated rejection; ADPKD, autosomal dominant polycystic kidney disease; AR, acute rejection; BMI, body mass index; DGF, delayed graft function; EIA, enzyme immunoassays; IIF, indirect immunofluorescence; KRT, kidney replacement therapy; KT, kidney transplantation; PRA, panel reactive antibody; TCMR, T-cell mediated rejection.
"-" means not applicable. When there was not enough data in some subgroup, Cox regression model could not converge (result is given as "Inf.")

Supplementary Table S9 – Distribution of delays between diagnosis, ESKD, waitlisting and kidney transplantation

Supplementary Table S1 – Comparison of AAV-GN patients according to ANCA status at KT

	N	**Negative**, N = 68	**Positive in at least 1 test (IIF or EIA)**, N = 99	**p-value**
**Baseline characteristics**				
Male sex, n (%)	167	46 (68%)	66 (67%)	0.9
BMI (kg/m²)	167	25.9 (3.5)	24.2 (4.3)	**0.007**
Hypertension, n (%)	167	57 (84%)	86 (87%)	0.6
Diabetes, n (%)	167	8 (12%)	12 (12%)	>0.9
**Presentation at AAV-GN diagnosis**				
Age (years)	161	54 (13)	52 (13)	0.3
Kidney involvement, n (%)	167	68 (100%)	99 (100%)	
Creatinine (µmol/L)	111	610 (420)	483 (344)	0.1
Need for KRT within 30 days, n (%)	131	30 (57%)	29 (37%)	**0.028**
Proteinuria (g/g)	98	1.87 (1.26)	3.46 (3.48)	**0.002**
Hematuria, n (%)	104	43 (98%)	55 (92%)	0.4
Kidney biopsy, n (%)	148	56 (92%)	84 (97%)	0.3
Lung involvement, n (%)	152	24 (38%)	31 (35%)	0.7
Heart involvement, n (%)	151	4 (6.5%)	1 (1.1%)	0.2
Neurological involvement, n (%)	151	5 (8.1%)	8 (9.0%)	0.8
ENT involvement, n (%)	152	11 (18%)	21 (23%)	0.4
**Immunological findings**				
Presence of ANCA, n (%)	158	62 (97%)	92 (98%)	>0.9
Anti-PR3	146	22 (37%)	16 (19%)	**0.014**
Anti-MPO	146	38 (64%)	70 (80%)	**0.03**
**Therapeutic management of AAV-GN**				
Induction remission therapy				
Plasma exchange, n (%)	150	26 (47%)	30 (32%)	0.055
Methylprednisolone pulses, n (%)	149	59 (97%)	78 (89%)	0.12
Prednisone, n (%)	158	59 (94%)	89 (94%)	>0.9
Cyclophosphamide, n (%)	156	54 (86%)	69 (74%)	0.084
Rituximab, n (%)	155	6 (9.5%)	12 (13%)	0.5
Maintenance therapy				
Prednisone, n (%)	133	44 (80%)	69 (88%)	0.2
Cyclophosphamide, n (%)	145	8 (14%)	9 (10%)	0.4
Rituximab, n (%)	146	14 (25%)	18 (20%)	0.5
Azathioprine, n (%)	146	19 (34%)	35 (39%)	0.5
Mycophenolic acid, n (%)	146	12 (21%)	16 (18%)	0.6
**Outcomes before KT**				
AAV relapse	163	19 (29%)	29 (30%)	0.9
Preemptive transplantation	167	6 (8.8%)	6 (6.1%)	0.6
**Status at KT**				
Recipient age (years)	167	61 (11)	59 (11)	0.3
First transplantation	167	64 (94%)	93 (94%)	>0.9
Calculated PRA	135	14 (29)	16 (28)	0.8
**Transplantation procedure**				
Donor age (years)	158	61 (13)	58 (15)	0.2
Deceased donor, n (%)	167	58 (85%)	91 (92%)	0.2
ABO incompatibility, n (%)	144	0 (0%)	0 (0%)	>0.9
Donor creatinine (µmol/L)	148	77 (51)	81 (50)	0.6
Cold ischemia time (hours)	167	13 (7)	14 (7)	0.6
HLA-mismatches (total)	166	4.34 (1.55)	4.76 (1.56)	0.094
HLA-A	166	1.21 (0.64)	1.28 (0.66)	0.5
HLA-B	166	1.39 (0.58)	1.45 (0.59)	0.5
HLA-DR	166	1.09 (0.73)	1.11 (0.60)	0.8
HLA-DQ	149	0.81 (0.75)	0.95 (0.67)	0.3
**Immunosuppressive regimen**				
Induction therapy				
Anti-thymocyte globulin, n (%)	167	23 (34%)	32 (32%)	0.8
Basiliximab, n (%)	167	45 (66%)	65 (66%)	>0.9
Prednisone, n (%)	165	64 (94%)	94 (97%)	0.4
Maintenance regimen				
Calcineurin inhibitors, n (%)	167	68 (100%)	99 (100%)	>0.9
Mycophenolic acid, n (%)	167	63 (93%)	92 (93%)	>0.9
Prednisone, n (%)	163	67 (99%)	86 (91%)	**0.046**
mTOR inhibitors, n (%)	162	17 (27%)	19 (19%)	0.2
Azathioprine, n (%)	165	6 (9.0%)	8 (8.2%)	0.9
**Outcomes after KT**				
Follow-up duration (months)	167	54 (43)	63 (49)	0.2
DGF, n (%)	166	12 (18%)	19 (19%)	0.8
Allograft failure, n (%)	167	10 (15%)	17 (17%)	0.7
Relapses				
All, n (%)	167	2 (2.9%)	11 (11%)	0.053
With kidney involvement, n (%)	167	2 (2.9%)	8 (8.1%)	0.2
Rejection, n (%)	165	15 (22%)	14 (14%)	0.2
Acute rejection (AR), n (%)	165	15 (22%)	14 (14%)	0.2
TCMR, n (%)	166	11 (16%)	7 (7.1%)	0.057
ABMR, n (%)	166	3 (4.5%)	6 (6.1%)	0.7
Mixed AR, n (%)	166	1 (1.5%)	1 (1.0%)	>0.9
Chronic rejection	166	4 (6.0%)	3 (3.0%)	0.4
Death	166	15 (22%)	24 (24%)	0.8

Supplementary Table S2 – Factors associated with DGF (in the AAV-GN cohort)

	Univariable					Multivariable (simplified)				
**Baseline characteristics**	**N**	**Event N**	**OR**[1]	**95% CI**[1]	**p-value**	**N**	**Event N**	**OR**[1]	**95% CI**[1]	**p-value**
Male sex (vs female)	204	37	1.26	0.59, 2.82	0.6					
BMI (kg/m²)	204	37	1.07	0.98, 1.16	0.13					
Hypertension (vs no)	202	37	1.54	0.55, 5.49	0.4					
Diabetes (vs no)	204	37	1.71	0.58, 4.49	0.3					
**Presentation at AAV-GN diagnosis**										
Age (years)	198	36	1.02	0.99, 1.06	0.15					
Kidney involvement (vs no)	201	36	Inf.	Inf.	-					
Lung involvement (vs no)	183	33	0.80	0.34, 1.76	0.6					
Heart involvement (vs no)	182	33	0.90	0.05, 5.83	>0.9					
Neurological involvement (vs no)	182	33	0.81	0.12, 3.21	0.8					
ENT involvement (vs no)	183	33	0.99	0.37, 2.39	>0.9					
**Immunological findings**										
Presence of ANCA (vs no)	191	34	0.21	0.02, 1.77	0.12					
Anti-PR3 (vs anti-MPO)	174	31	0.91	0.36, 2.15	0.8					
**Outcomes before KT**										
Relapse	197	36	0.35	0.11, 0.88	**0.039**	173	33	0.23	0.05, 0.70	**0.021**
Preemptive transplantation	204	37	0.36	0.02, 1.91	0.3					
**Kidney transplantation**										
**Status at KT**										
Age (years)	204	37	1.02	0.99, 1.06	0.2					
First transplantation	204	37	0.64	0.21, 2.39	0.5					
Calculated PRA	155	27	1.00	0.99, 1.01	>0.9					
**ANCA status at KT**										
According to IIF only - positive, n (%)	142	27	1.33	0.57, 3.19	0.5					
According to EIA only - positive, n (%)	120	23	1.44	0.58, 3.68	0.4					
According to at least one test (IIF or EIA) - at least one positive	166	31	1.09	0.49, 2.48	0.8					
According to both test (IIF and EIA)	97	19								
Double negative			Ref.	-	-					
Discordant			0.66	0.13, 2.80	0.6					
IIF positive & EIA negative			0.81	0.15, 3.47	0.8					
IIF negative & EIA positive			Inf.	Inf.	-					
Double positive			1.79	0.58, 5.89	0.3					
**Transplantation procedure**										
Donor age (years)	194	33	1.00	0.97, 1.03	>0.9					
Deceased donor (vs no)	204	37	Inf.	Inf.	-					
Donor creatinine (per 50 µmol/L increment)	178	34	1.29	0.92, 1.82	0.13					
Cold ischemia time (hours)	203	37	1.07	1.01, 1.13	**0.016**	173	33	1.07	1.00, 1.14	**0.043**
HLA-mismatches (total)	204	37	0.85	0.69, 1.06	0.15					
HLA-A	203	37	0.88	0.51, 1.53	0.6					
HLA-B	203	37	1.01	0.56, 1.88	>0.9					
HLA-DR	203	37	0.75	0.44, 1.28	0.3					
HLA-DQ	180	34	0.47	0.26, 0.82	**0.010**	173	33	0.51	0.28, 0.91	**0.027**
**Immunosuppressive regimen**										
Induction therapy										
Anti-thymocyte globulin (vs no)	204	37	0.77	0.35, 1.64	0.5					
Basiliximab (vs no)	204	37	1.17	0.56, 2.56	0.7					
Prednisone (vs no)	202	36	0.53	0.11, 3.79	0.5					
Maintenance regimen										
Calcineurin inhibitors (vs no)	204	37	Inf.	Inf.	-					
Mycophenolic acid (vs no)	204	37	1.23	0.31, 8.20	0.8					
Prednisone (vs no)	199	37	0.33	0.10, 1.16	0.067					
mTOR inhibitors (vs no)	200	35	0.46	0.15, 1.16	0.13					
Azathioprine (vs no)	202	37	1.03	0.23, 3.42	>0.9					

Supplementary Table S3 – Factors associated with graft failure (in the whole cohort)

	Univariable					Multivariable (simplified)				
	**N**	**Event N**	**HR**[1]	**95% CI**[1]	**p-value**	**N**	**Event N**	**HR**[1]	**95% CI**[1]	**p-value**
AAV-GN (vs CTRL)	617	79	1.44	0.91, 2.28	0.11	571	71	1.55	0.95, 2.50	0.077
**Baseline characteristics**										
Male sex (vs female)	617	79	1.43	0.87, 2.37	0.2					
BMI (kg/m²)	614	79	0.96	0.91, 1.01	0.2					
Hypertension (vs no)	592	74	0.66	0.36, 1.20	0.2					
Diabetes (vs no)	616	78	1.04	0.56, 1.93	0.9					
**Kidney transplantation**										
**Status at KT**										
Age (years)	617	79	1.03	1.01, 1.05	**0.012**					
First transplantation	617	79	0.66	0.36, 1.23	0.2					
Preemptive transplantation	617	79	0.36	0.13, 0.98	**0.045**					
Calculated PRA	458	46	1.00	1.00, 1.01	0.3					
**Transplantation procedure**										
Donor age (years)	590	73	1.04	1.02, 1.05	**<0.001**	571	71	1.04	1.02, 1.06	**<0.001**
Deceased donor (vs no)	617	79	2.61	0.64, 10.6	0.2					
Donor creatinine (per 50 µmol/L increment)	541	63	0.86	0.61, 1.21	0.4					
Cold ischemia time (hours)	605	76	1.02	0.99, 1.05	0.2					
HLA-mismatches (total)	617	79	0.97	0.85, 1.11	0.7					
HLA-A	611	76	1.07	0.78, 1.48	0.7					
HLA-B	611	76	1.17	0.81, 1.68	0.4					
HLA-DR	611	76	1.03	0.74, 1.43	0.9					
HLA-DQ	529	69	0.81	0.59, 1.11	0.2					
**Immunosuppressive regimen**										
Induction therapy										
Anti-thymocyte globulin (vs no)	613	77	0.98	0.61, 1.59	>0.9					
Basiliximab (vs no)	613	77	1.00	0.63, 1.60	>0.9					
Prednisone (vs no)	612	76	1.22	0.30, 4.97	0.8					
Maintenance regimen										
Calcineurin inhibitors (vs no)	616	78	0.22	0.05, 0.91	**0.036**					
Mycophenolic acid (vs no)	614	77	0.32	0.15, 0.69	**0.004**					
Prednisone (vs no)	597	76	0.85	0.49, 1.45	0.5					
mTOR inhibitors (vs no)	608	78	0.95	0.53, 1.69	0.9					
Azathioprine (vs no)	608	77	2.06	0.99, 4.29	0.053	571	71	2.56	1.20, 5.45	**0.015**
**Outcomes after KT**										
DGF (vs no)	613	79	3.08	1.95, 4.86	**<0.001**	571	71	2.28	1.38, 3.77	**0.001**
Rejection (vs no)	608	78	3.18	2.03, 4.97	**<0.001**	571	71	2.85	1.77, 4.59	**<0.001**
Acute rejection (AR) (vs no)	607	77	2.83	1.79, 4.45	**<0.001**					
TCMR (vs no)	598	76	1.25	0.71, 2.22	0.4					
ABMR (vs no)	599	76	6.37	3.49, 11.6	**<0.001**					
Mixed AR (vs no)	615	79	4.00	1.61, 9.93	**0.003**					
Chronic rejection	600	77	4.64	2.63, 8.18	**<0.001**					

Supplementary Table S4 – Description of AAV-GN patients according to relapsing status

	N	No relapse, N = 191	Relapse, N = 15	p-value
**Baseline characteristics**				
Male sex, n (%)	206	124 (65%)	13 (87%)	0.086
BMI (kg/m²)	206	24.2 (21.7, 27.7)	24.9 (23.6, 27.3)	0.3
Hypertension, n (%)	204	163 (86%)	11 (73%)	0.2
Diabetes, n (%)	206	21 (11%)	2 (13%)	0.7
**Presentation at AAV-GN diagnosis**				
Age (years)	200	54 (48, 61)	51 (47, 56)	0.3
Kidney involvement, n (%)	203	188 (100%)	15 (100%)	
Creatinine (µmol/L)	133	450 (300, 642)	423 (315, 782)	>0.9
Need for KRT within 30 days, n (%)	160	66 (45%)	6 (50%)	0.7
Proteinuria (g/g)	119	1.80 (1.00, 3.19)	1.90 (0.83, 3.55)	0.7
Hematuria, n (%)	126	111 (96%)	9 (90%)	0.4
Kidney biopsy, n (%)	184	161 (94%)	11 (92%)	0.6
Lung involvement, n (%)	185	59 (34%)	5 (38%)	0.8
Heart involvement, n (%)	184	6 (3.5%)	0 (0%)	>0.9
Neurological involvement, n (%)	184	11 (6.4%)	2 (15%)	0.2
ENT involvement, n (%)	185	35 (20%)	4 (29%)	0.5
**Immunological findings**	184	55 (32%)	7 (50%)	0.2
Presence of ANCA, n (%)	193	176 (99%)	13 (87%)	**0.031**
Anti-PR3, n (%)	177	46 (28%)	3 (20%)	0.8
Anti-MPO, n (%)	176	117 (73%)	11 (73%)	>0.9
**Therapeutic management of AAV-GN**				
Induction remission therapy				
Plasma exchange, n (%)	185	59 (35%)	5 (33%)	>0.9
Methylprednisolone pulses, n (%)	183	156 (93%)	12 (80%)	0.11
Prednisone, n (%)	195	170 (94%)	13 (87%)	0.2
Cyclophosphamide, n (%)	192	139 (79%)	11 (73%)	0.7
Rituximab, n (%)	191	18 (10%)	1 (6.7%)	>0.9
Maintenance therapy				
Prednisone, n (%)	160	124 (84%)	10 (77%)	0.4
Cyclophosphamide, n (%)	177	16 (9.8%)	3 (21%)	0.2
Rituximab, n (%)	178	34 (21%)	1 (7.1%)	0.3
Azathioprine, n (%)	178	60 (37%)	5 (36%)	>0.9
Mycophenolic acid, n (%)	178	27 (16%)	3 (21%)	0.7
**Outcomes before KT**				
Relapse	199	51 (28%)	5 (33%)	0.8
KRT for ESKD (non preemptive)	206	177 (93%)	14 (93%)	>0.9
**Status at KT**				
Age (years)	206	60 (54, 67)	59 (56, 62)	0.3
First transplantation	206	177 (93%)	15 (100%)	0.6
Calculated PRA	157	0 (0, 25)	0 (0, 0)	**0.049**
**ANCA status at KT**				
According to IIF only - positive, n (%)	143	68 (51%)	8 (80%)	0.1
According to EIA only - positive, n (%)	120	53 (49%)	6 (50%)	>0.9
According to at least one test (IIF or EIA)	167			0.053
Negative, n (%)		66 (43%)	2 (15%)	
At least one positive, n (%)		88 (57%)	11 (85%)	
According to both test (IIF and EIA)	97			0.4
Double negative, n (%)		33 (38%)	2 (22%)	
Discordant, n (%)		21 (24%)	4 (44%)	
IIF positive & EIA negative, n (%)		17 (19%)	4 (44%)	
IIF negative & EIA positive, n (%)		4 (4.5%)	0 (0%)	
Double positive, n (%)		34 (39%)	3 (33%)	
**Transplantation procedure**				
Donor age (years)	195	61 (51, 69)	56 (52, 66)	0.4
Deceased donor, n (%)	206	171 (90%)	15 (100%)	0.4
Donor creatinine (µmol/L)	180	68 (54, 86)	69 (65, 73)	0.7
Cold ischemia time (hours)	205	14 (9, 18)	14 (11, 16)	>0.9
HLA-mismatches (total)	206	5.00 (4.00, 6.00)	5.00 (4.00, 5.50)	0.6
HLA-A	205	1.00 (1.00, 2.00)	1.00 (1.00, 2.00)	>0.9
HLA-B	205	1.00 (1.00, 2.00)	2.00 (1.00, 2.00)	0.8
HLA-DR	205	1.00 (1.00, 2.00)	1.00 (1.00, 2.00)	0.8
HLA-DQ	180	1.00 (0.00, 1.00)	1.00 (0.50, 1.00)	0.9
**Immunosuppressive regimen**				
Induction therapy				
Anti-thymocyte globulin, n (%)	206	68 (36%)	3 (20%)	0.2
Basiliximab, n (%)	206	122 (64%)	11 (73%)	0.5
Prednisone, n (%)	204	184 (97%)	13 (93%)	0.4
Maintenance regimen				
Calcineurin inhibitors, n (%)	206	191 (100%)	15 (100%)	
Mycophenolic acid, n (%)	206	180 (94%)	13 (87%)	0.2
Prednisone, n (%)	201	174 (94%)	14 (93%)	>0.9
mTOR inhibitors, n (%)	201	50 (27%)	0 (0%)	**0.025**
Azathioprine, n (%)	203	14 (7.4%)	2 (13%)	0.3
**Outcomes after KT**				
Follow-up duration (months)	206	50 (26, 81)	66 (40, 140)	0.2
DGF, n (%)	204	35 (19%)	2 (13%)	>0.9
Allograft failure, n (%)	206	21 (11%)	9 (60%)	**<0.001**
Relapses with kidney involvement, n (%)	206	-	12 (80%)	-
Rejection, n (%)	204	33 (17%)	6 (40%)	**0.044**
Acute rejection (AR), n (%)	204	32 (17%)	6 (40%)	**0.039**
TCMR, n (%)	205	20 (11%)	2 (13%)	0.7
ABMR, n (%)	205	8 (4.2%)	3 (20%)	**0.037**
Mixed AR, n (%)	205	4 (2.1%)	1 (6.7%)	0.3
Chronic rejection	205	6 (3.2%)	2 (13%)	0.11
Death	205	45 (24%)	5 (33%)	0.4

Supplementary Table S5 – Description of each AAV-GN relapse

		AAV-GN diagnosis							Kidney transplantation							Outcomes after KT							
Patient #	Sex	Year of diagnosis	Age at diagnosis (years)	Disease presentation at diagnosis	ANCA subtype	Initial management: induction	Initial management: maintenance	Relapse before KT	Year of KT	Time from diag. to KT (months)	Time from ESKD to KT (months)	Age at KT (years)	Immunosuppressive regimen post KT	ANCA status at KT (IIF or EIA)	ANCA status at KT (IIF and EIA)	Time from KT to relapse (months)	Relapse: kidney involving	Relapse: other organs involved	ANCA status at relapse (IIF or EIA)	Relapse: treatment	Graft failure	Time from relapse to graft failure (months)	Time from KT to graft failure (or last follow-up) (months)
1	M	2005	54	Kidney only	MPO	-	-	No	2008	32	31	56	BSX, Ster, CNI, MMF	-	-	36	Yes	No	Positive	Increase Ster, increase CNI, RTX	Yes	58	95
2	M	1992	47	Kidney +	PR3	PLEX, Ster, CYC	Ster	Yes	2012	236	26	66	BSX, CNI, MMF	Positive	Discordance	85	No	Yes	Positive	RTX	No	-	146
3	M	2013	48	Kidney +	MPO	PLEX, Ster, CYC	Ster, RTX	Yes	2021	97	72	56	ATG, Ster, CNI, MMF	Positive	-	10 days	Yes	No	Positive	PLEX, CYC	Yes	1	1
4	F	2004	52	Kidney +	MPO	Ster, CYC	Ster, AZA	No	2007	39	36	55	BSX, Ster, CNI, MMF	-	-	55	Yes	Yes	Positive	Increase Ster, CYC, CNI, PLEX then AZA	No	-	92
5	M	2005	57	Kidney +	MPO	Ster	Ster	No	2006	16	15	59	ATG, Ster, CNI, MMF	Positive	Discordance	100	Yes	Yes	Positive	Increase Ster, continue CNI	Yes	58	159
6	M	-	-	Kidney only	MPO	PLEX, Ster, CYC	Ster, AZA	No	2008		12	59	BSX, Ster, CNI, MMF	Positive	Positive	21 days	Yes	Yes	Positive	Increase Ster, Increase MMF then CYC, Lower CNI, IVIg	No	-	45
7	M	2013	44	Kidney +	MPO	PLEX, Ster, CYC	Ster, CYC/AZA/MMF	No	2015	31	20	47	BSX, Ster, CNI, MMF	Positive	Positive	44	Yes	No	Positive	Increase Ster, RTX, CNI	Yes	18	62
8	M	2006	53	Kidney +	MPO	Ster, CYC	Ster, CYC/AZA	Yes	2008	26	17	56	BSX, Ster, CNI, AZA	Positive	Positive	52	Yes	No	Positive	PLEX, RTX, Increase AZA and Ster	Yes	26	79
9	M	1993	47	Kidney +	MPO	Ster, CYC	Ster, CYC	No	2006	158	158	60	ATG, Ster, CNI, MMF	Positive	Discordance	1	Yes	No	Positive	RTX, IVIg	No	-	170
10	F	2010	61	Kidney +	PR3	Ster, CYC	AZA	No	2017	89	89	69	BSX, Ster, CNI, AZA	Positive	Discordance	65	Yes	No	Positive	Increase Ster, RTX	No	-	66
11	M	2002	43	Kidney only	MPO	-	-	No	2013	138	70	54	BSX, Ster, CNI, MMF	Negative	Negative	40	Yes	No	-	PLEX, Ster, IV CNI	Yes	3	44
12	M	2013	58	Kidney +	Negative	Ster, CYC	Ster, MMF	No	2017	41	41	62	BSX, Ster, CNI, MMF	Negative	Negative	7	Yes	No	-	Increase Ster, CYC, CNI, PLEX then AZA	No	-	8
13	M	2006	47	Kidney +	MPO	Ster, CYC	Ster, MMF	Yes	2009	30	13	49	Ster, CNI, MMF	Positive	-	25	No	Yes	Positive	Increase Ster, CYC then AZA	Yes	Relapse after ESKD	15
14	M	1996	50	Kidney +	PR3	Ster, CYC	-	Yes	2008	141	0	62	BSX, Ster, CNI, MMF	Positive	-	45	No	Yes	Positive	Increase Ster, RTX	Yes	82	128
15	M	2019	61	Kidney +	MPO	Ster, CYC	Ster	No	2022	46	46	65	BSX, Ster, CNI, MMF	Positive	-	8 days	Yes	Yes	Positive	Increase Ster, PLEX, RTX + CYC	Yes	9 days	0

Supplementary Table S6 – Associated between ANCA status at KT, relapses and acute rejection (multivariable models) (in the AAV-GN cohort)

	Model 1 - IIF					Model 2 - EIA					Model 3 - IIF or EIA					Model 4 - IIF and EIA					Model 5 - IIF and EIA (bis)				
**Cox regression for relapse-free survival**	**N**	**Event N**	**HR**	**95% CI**	**p-value**	**N**	**Event N**	**HR**	**95% CI**	**p-value**	**N**	**Event N**	**HR**	**95% CI**	**p-value**	**N**	**Event N**	**HR**	**95% CI**	**p-value**	**N**	**Event N**	**HR**	**95% CI**	**p-value**
Presence of ANCA at AAV-GN diagnosis (vs no)	134	10	0.03	0.00, 0.20	**<0.001**	115	12	0.05	0.01, 0.31	**0.001**	158	13	0.04	0.01, 0.24	**<0.001**	92	9	0.03	0.00, 0.24	**<0.001**	92	9	0.03	0.00, 0.24	**<0.001**
ANCA status at transplantation																									
According to IIF only - positive	134	10	3.81	0.80, 18.2	0.094																				
According to EIA only - positive						115	12	1.35	0.41, 4.42	0.6															
According to at least one test (IIF or EIA) - at least one positive											158	13	4.17	0.91, 19.0	0.065										
According to both test (IIF and EIA)																92	9				92	9			
Double negative																		—	—				—	—	
Discordant																		3.34	0.59, 19.0	0.2					
IIF positive & EIA negative																							3.66	0.97, 13.8	0.055
IIF negative & EIA positive																							Inf.	Inf.	-
Double positive																		1.84	0.28, 11.9	0.5			1.79	0.44, 7.37	0.4

	Model 1 - IIF					Model 2 - EIA					Model 3 - IIF or EIA					Model 4 - IIF and EIA					Model 5 - IIF and EIA (bis)				
**Cox regression for acute rejection-free survival**	**N**	**Event N**	**HR**	**95% CI**	**p-value**	**N**	**Event N**	**HR**	**95% CI**	**p-value**	**N**	**Event N**	**HR**	**95% CI**	**p-value**	**N**	**Event N**	**HR**	**95% CI**	**p-value**	**N**	**Event N**	**HR**	**95% CI**	**p-value**
Presence of ANCA at AAV-GN diagnosis (vs no)	130	21	0.19	0.04, 0.87	**0.032**	112	24	0.31	0.07, 1.39	0.13	154	28	0.25	0.06, 1.07	0.062	89	17	0.28	0.06, 1.27	0.1	89	17	0.25	0.05, 1.16	0.076
KT maintenance with AZA (vs no)	130	21	3.73	1.34, 10.4	**0.012**	112	24	2.15	0.72, 6.40	0.2	154	28	2.6	0.97, 6.93	0.056	89	17	3.6	1.14, 11.4	**0.03**	89	17	4.04	1.25, 13.0	**0.019**
ANCA status at transplantation																									
According to IIF only - positive	130	21	0.31	0.12, 0.81	**0.016**																				
According to EIA only - positive						112	24	0.7	0.30, 1.63	0.4															
According to at least one test (IIF or EIA) - at least one positive											154	28	0.54	0.25, 1.15	0.11										
According to both test (IIF and EIA)																89	17				89	17			
Double negative																		—	—				—	—	
Discordant																		0.3	0.08, 1.08	0.065					
IIF positive & EIA negative																							0.2	0.04, 0.93	**0.04**
IIF negative & EIA positive																							2.15	0.26, 17.5	0.5
Double positive																		0.16	0.04, 0.74	**0.019**			0.16	0.04, 0.74	**0.019**

Supplementary Table S7 – Factors associated with acute rejection (in the whole cohort)

	Univariable					Multivariable (simplified)				
	**N**	**Event N**	**HR**[1]	**95% CI**[1]	**p-value**	**N**	**Event N**	**HR**[1]	**95% CI**[1]	**p-value**
AAV-GN (vs CTRL)	604	108	1.12	0.75, 1.66	0.6	578	102	1.07	0.71, 1.61	0.8
**Baseline characteristics**										
Male sex (vs female)	604	108	1.56	1.01, 2.42	**0.045**	578	102	1.59	1.01, 2.51	**0.044**
BMI (kg/m²)	601	108	1.01	0.97, 1.05	0.6					
Hypertension (vs no)	579	102	0.60	0.36, 1.00	**0.049**	578	102	0.58	0.34, 0.96	**0.035**
Diabetes (vs no)	603	108	0.93	0.56, 1.54	0.8					
**Kidney transplantation**										
**Status at KT**										
Age (years)	604	108	1.00	0.98, 1.01	0.7					
First transplantation	604	108	2.86	1.16, 7.04	**0.023**	578	102	2.38	0.96, 5.91	0.061
Preemptive transplantation	604	108	0.65	0.33, 1.29	0.2					
Calculated PRA	449	61	1.00	0.99, 1.00	0.3					
**Transplantation procedure**										
Donor age (years)	577	106	1.00	0.99, 1.02	0.8					
Deceased donor (vs no)	604	108	1.61	0.66, 3.96	0.3					
Donor creatinine (per 50 µmol/L increment)	528	95	0.90	0.68, 1.18	0.4					
Cold ischemia time (hours)	592	104	1.00	0.97, 1.03	0.9					
HLA-mismatches (total)	604	108	1.13	1.01, 1.27	**0.033**					
HLA-A	598	107	1.13	0.85, 1.50	0.4					
HLA-B	598	107	1.09	0.80, 1.47	0.6					
HLA-DR	598	107	1.28	0.97, 1.69	0.085					
HLA-DQ	516	97	1.24	0.94, 1.63	0.12					
**Immunosuppressive regimen**										
Induction therapy										
Anti-thymocyte globulin (vs no)	600	108	0.61	0.39, 0.96	**0.032**					
Basiliximab (vs no)	600	108	1.44	0.94, 2.21	0.092					
Prednisone (vs no)	599	107	0.89	0.28, 2.82	0.8					
Maintenance regimen										
Calcineurin inhibitors (vs no)	603	108	0.24	0.06, 0.96	**0.044**	578	102	0.22	0.05, 0.90	**0.036**
Mycophenolic acid (vs no)	601	107	0.74	0.30, 1.83	0.5					
Prednisone (vs no)	585	105	1.53	0.87, 2.69	0.14					
mTOR inhibitors (vs no)	595	106	1.37	0.88, 2.15	0.2					
Azathioprine (vs no)	595	105	1.82	0.92, 3.62	0.088					
**Outcomes after KT**										
DGF (vs no)	600	108	1.35	0.85, 2.14	0.2					

Supplementary Table S8 – Factors associated with death (in the whole cohort)

	Univariable					Multivariable (simplified)				
	**N**	**Event N**	**HR**[1]	**95% CI**[1]	**p-value**	**N**	**Event N**	**HR**[1]	**95% CI**[1]	**p-value**
AAV-GN (vs CTRL)	617	131	1.48	1.04, 2.10	**0.031**	616	131	1.48	1.03, 2.13	**0.034**
**Baseline characteristics**										
Male sex (vs female)	617	131	1.30	0.88, 1.90	0.2					
BMI (kg/m²)	614	131	1.00	0.96, 1.04	>0.9					
Hypertension (vs no)	592	122	0.86	0.52, 1.44	0.6					
Diabetes (vs no)	616	131	2.31	1.57, 3.40	**<0.001**	616	131	1.79	1.20, 2.68	**0.004**
**Kidney transplantation**										
**Status at KT**										
Age (years)	617	131	1.09	1.06, 1.11	**<0.001**	616	131	1.08	1.06, 1.10	**<0.001**
First transplantation	617	131	1.02	0.59, 1.77	>0.9					
Preemptive transplantation	617	131	0.80	0.46, 1.40	0.4					
Calculated PRA	458	88	1.00	1.0, 1.01	0.8					
**Transplantation procedure**										
Donor age (years)	590	126	1.04	1.03, 1.06	**<0.001**					
Deceased donor (vs no)	617	131	1.35	0.60, 3.07	0.5					
Donor creatinine (per 50 µmol/L increment)	540	117	0.91	0.72, 1.15	0.4					
Cold ischemia time (hours)	605	128	1.01	0.99, 1.04	0.2					
HLA-mismatches (total)	617	131	1.07	0.96, 1.19	0.2					
HLA-A	611	130	1.28	0.99, 1.65	0.056					
HLA-B	611	130	1.02	0.78, 1.34	0.9					
HLA-DR	611	130	1.04	0.81, 1.33	0.8					
HLA-DQ	529	113	1.05	0.83, 1.34	0.7					
**Immunosuppressive regimen**										
Induction therapy										
Anti-thymocyte globulin (vs no)	613	131	1.01	0.71, 1.46	>0.9					
Basiliximab (vs no)	613	131	1.15	0.80, 1.65	0.4					
Prednisone (vs no)	612	130	0.80	0.33, 1.95	0.6					
Maintenance regimen										
Calcineurin inhibitors (vs no)	616	131	1.22	0.17, 8.74	0.8					
Mycophenolic acid (vs no)	614	131	1.11	0.41, 3.01	0.8					
Prednisone (vs no)	597	125	1.20	0.75, 1.91	0.5					
mTOR inhibitors (vs no)	609	129	0.98	0.63, 1.52	>0.9					
Azathioprine (vs no)	608	129	0.95	0.44, 2.04	0.9					
**Outcomes after KT**										
DGF (vs no)	613	128	1.37	0.90, 2.08	0.15					
Allograft failure (vs no)	617	131	0.97	0.59, 1.60	>0.9					
Rejection (vs no)	608	129	1.05	0.70, 1.57	0.8					
Acute rejection (AR) (vs no)	607	130	0.99	0.66, 1.50	>0.9					
TCMR (vs no)	598	128	0.81	0.49, 1.34	0.4					
ABMR (vs no)	599	128	1.37	0.64, 2.94	0.4					
Mixed AR (vs no)	615	130	1.24	0.39, 3.90	0.7					
Chronic rejection	600	129	1.19	0.61, 2.35	0.6					

Supplementary Table S9 – Distribution of delays between diagnosis, ESKD, waitlisting and kidney transplantation

Delays	N	AAV-GN, N = 206	CTRL, N = 412	p-value
Follow-up (time from KT to last visit)	618	52 (26, 84)	60 (30, 104)	0.051
**AAV-GN diagnosis to ESKD (months)**	185	17 (1, 58)	-	
More than 6 months	185	112 (61%)	-	
More than 12 months	185	99 (54%)	-	
More than 24 months	185	84 (45%)	-	
3 categories	185			
<12 months		86 (46%)	-	
12-36 months		32 (17%)	-	
>36 months		67 (36%)	-	
5 categories	185			
<6 months		73 (39%)	-	
6-12 months		13 (7.0%)	-	
12-24 months		15 (8.1%)	-	
24-36 months		17 (9.2%)	-	
>36 months		67 (36%)	-	
**AAV-GN diagnosis to KT (months)**	200	57 (36, 104)	-	
More than 6 months	200	199 (100%)	-	
More than 12 months	200	198 (99%)	-	
More than 24 months	200	181 (91%)	-	
3 categories	200			
<12 months		2 (1.0%)	-	
12-36 months		48 (24%)	-	
>36 months		150 (75%)	-	
5 categories	200			
<6 months		1 (0.5%)	-	
6-12 months		1 (0.5%)	-	
12-24 months		17 (8.5%)	-	
24-36 months		31 (16%)	-	
>36 months		150 (75%)	-	
**AAV-GN diagnosis to waitlisting**	184	42 (20, 81)	-	
More than 6 months	184	181 (98%)	-	
More than 12 months	184	170 (92%)	-	
More than 24 months	184	129 (70%)	-	
3 categories	184			
<12 months		14 (7.6%)	-	
12-36 months		70 (38%)	-	
>36 months		100 (54%)	-	
5 categories	184			
<6 months		3 (1.6%)	-	
6-12 months		11 (6.0%)		
12-24 months		41 (22%)		
24-36 months		29 (16%)	-	
>36 months		100 (54%)	-	
**ESKD to KT**	617	30 (18, 46)	25 (9, 47)	**0.023**
More than 6 months	617	186 (90%)	329 (80%)	**0.001**
More than 12 months	617	179 (87%)	290 (71%)	**<0.001**
More than 24 months	617	131 (64%)	219 (53%)	**0.015**
3 categories	617			**<0.001**
<12 months		27 (13%)	121 (29%)	
12-36 months		99 (48%)	141 (34%)	
>36 months		80 (39%)	149 (36%)	
5 categories	617			**<0.001**
<6 months		20 (9.7%)	82 (20%)	
6-12 months		7 (3.4%)	39 (9.5%)	
12-24 months		48 (23%)	71 (17%)	
24-36 months		51 (25%)	70 (17%)	
>36 months		80 (39%)	149 (36%)	
**ESKD to waitlisting**	501	14 (7, 22)	7 (-1, 18)	**<0.001**
More than 6 months	501	138 (78%)	186 (57%)	**<0.001**
More than 12 months	501	112 (63%)	118 (36%)	**<0.001**
More than 24 months	501	39 (22%)	60 (19%)	0.3
3 categories	501			**<0.001**
<12 months		65 (37%)	206 (64%)	
12-36 months		94 (53%)	81 (25%)	
>36 months		18 (10%)	37 (11%)	
5 categories	501			**<0.001**
<6 months		39 (22%)	138 (43%)	
6-12 months		26 (15%)	68 (21%)	
12-24 months		73 (41%)	58 (18%)	
24-36 months		21 (12%)	23 (7.1%)	
>36 months		18 (10%)	37 (11%)	
**Waitlisting to KT**	566	14 (7, 23)	17 (8, 31)	**0.044**
More than 6 months	566	152 (80%)	313 (83%)	0.3
More than 12 months	566	116 (61%)	237 (63%)	0.6
More than 24 months	566	47 (25%)	131 (35%)	**0.014**
3 categories	566			0.3
<12 months		74 (39%)	139 (37%)	
12-36 months		90 (47%)	166 (44%)	
>36 months		26 (14%)	71 (19%)	
5 categories	566			0.11
<6 months		38 (20%)	63 (17%)	
6-12 months		36 (19%)	76 (20%)	
12-24 months		69 (36%)	106 (28%)	
24-36 months		21 (11%)	60 (16%)	
>36 months		26 (14%)	71 (19%)	
